# Reconstruction of gene regulatory networks reveals chromatin remodelers and key transcription factors in tumorigenesis

**DOI:** 10.1186/s13073-016-0310-3

**Published:** 2016-05-19

**Authors:** Valeriya Malysheva, Marco Antonio Mendoza-Parra, Mohamed-Ashick M. Saleem, Hinrich Gronemeyer

**Affiliations:** Institut de Génétique et de Biologie Moléculaire et Cellulaire (IGBMC), Equipe Labellisée, Ligue Contre le Cancer, Centre National de la Recherche Scientifique UMR 7104, Institut National de la Santé et de la Recherche Médicale U964, University of Strasbourg, Illkirch, France

## Abstract

**Background:**

Alterations in genetic and epigenetic landscapes are known to contribute to the development of different types of cancer. However, the mechanistic links between transcription factors and the epigenome which coordinate the deregulation of gene networks during cell transformation are largely unknown.

**Methods:**

We used an isogenic model of stepwise tumorigenic transformation of human primary cells to monitor the progressive deregulation of gene networks upon immortalization and oncogene-induced transformation. We applied a systems biology approach by combining transcriptome and epigenome data for each step during transformation and integrated transcription factor–target gene associations in order to reconstruct the gene regulatory networks that are at the basis of the transformation process.

**Results:**

We identified 142 transcription factors and 24 chromatin remodelers/modifiers (CRMs) which are preferentially associated with specific co-expression pathways that originate from deregulated gene programming during tumorigenesis. These transcription factors are involved in the regulation of divers processes, including cell differentiation, the immune response, and the establishment/modification of the epigenome. Unexpectedly, the analysis of chromatin state dynamics revealed patterns that distinguish groups of genes which are not only co-regulated but also functionally related. Decortication of transcription factor targets enabled us to define potential key regulators of cell transformation which are engaged in RNA metabolism and chromatin remodeling.

**Conclusions:**

We reconstructed gene regulatory networks that reveal the alterations occurring during human cellular tumorigenesis. Using these networks we predicted and validated several transcription factors as key players for the establishment of tumorigenic traits of transformed cells. Our study suggests a direct implication of CRMs in oncogene-induced tumorigenesis and identifies new CRMs involved in this process. This is the first comprehensive view of the gene regulatory network that is altered during the process of stepwise human cellular tumorigenesis in a virtually isogenic system.

**Electronic supplementary material:**

The online version of this article (doi:10.1186/s13073-016-0310-3) contains supplementary material, which is available to authorized users.

## Background

During the past decade great progress has been made in identifying landscapes of genetic alterations which act at different gene regulatory levels and lead to the development of numerous cancer phenotypes. While much is known about altered signaling, recent studies have shown that the epigenomes of cancer cells can also dramatically deviate from those of the corresponding normal cells. However, little is known about the global deregulation of the transcriptome and epigenetic landscapes, as well as their crosstalk during the multistep process of cell transformation.

**Fig. 1:**
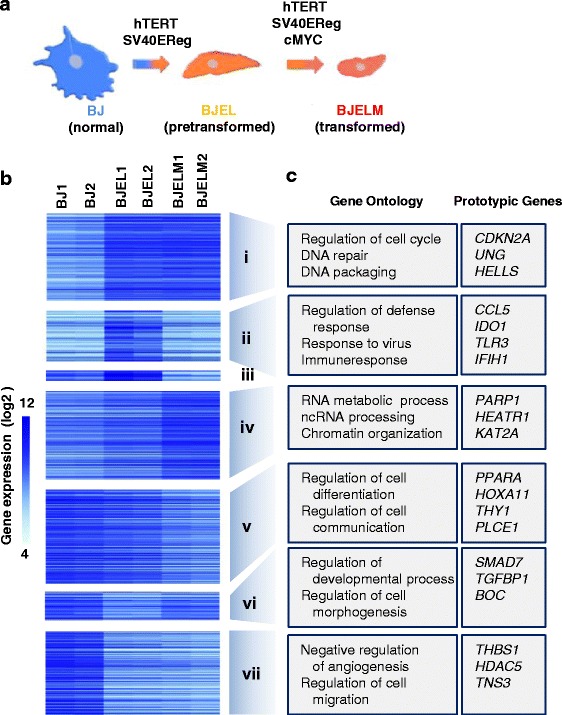
Transcriptional analysis of the stepwise cell transformation process. **a** BJ stepwise transformation cell model system. **b** Changes in the expression rate of differentially expressed genes (DEGs) in normal, immortalized, and transformed cells. **c** Biological process-based Gene Ontology analysis (performed with DAVID, *p* < 0.05; Additional file 2: Figure S2) for each co-regulated group of genes (co-expression pathways i to vii) and prototypic genes

The deregulatory processes that ultimately turn a normal cell into a tumor cell are conceptually well understood and have been described as “hallmarks of cancer” [1]. At the same time, the sequencing of cancer genomes provided an encyclopedia of somatic mutations, revealing the difficulty of working with primary human cancer cells that carry a small number of “driver” and a high number of variable “passenger” mutations [2]. To reduce this complexity and ensure cell-to-cell comparability, a stepwise human cellular transformation model [3] was chosen for the current study. In this model primary human cells (BJ) were first immortalized and pre-transformed into BJEL cells by the introduction of hTERT (the catalytic subunit of telomerase) and the large T and small t-antigen of the SV40 early region. The full transformation into bona fide tumor cells was achieved by overexpression of the c-*MYC* oncogene (Fig. 1a). The experimental advantage of this system is that normal, immortalized, and tumor cells are near isogenic, as revealed by single-nucleotide polymorphism (SNP) analysis (Additional file 1: Figure S1), such that data obtained for the pre-transformed and cancer cell can be accurately compared with the normal counterpart.

Epigenetic modifications comprising both DNA methylation and post-translational histone modifications or histone variants have been shown to affect transcription regulation. Different methylation patterns of lysine residues of histone H3 are widely used markers to describe the active and silenced states of transcription at the corresponding chromatin loci [4]. However, we know very little about how this regulation is altered during the process of tumorigenesis. The current study is among the first to reveal the interplay between the epigenome and transcriptome in a stepwise tumorigenesis system; it generates a working basis for understanding how this interplay is deregulated in a cellular model of human cancer. Here we addressed the following questions: (i) how are the global patterns of gene expression and chromatin organization changed; (ii) how are these levels coordinated during tumorigenesis; and (iii) what is the regulatory role of chromatin remodelers.

## Methods

### Cell culture

Primary human diploid BJ foreskin fibroblasts were obtained from the American Type Culture Collection (ATCC). Genetically defined cells of the BJ stepwise system (BJ and BJEL) were generously provided by Drs Hahn and Weinberg. BJELM cells were produced previously in our laboratory by retroviral transfection of the BJEL cell with pBabe-MYC-ER [5]. Cells were cultured in monolayer conditions in Dulbecco’s modified Eagle’s medium (DMEM)/M199 (4:1) (with 1 g/l glucose) supplemented with 10 % heat inactivated fetal calf serum (FCS) and gentamicin. The medium for BJEL was supplemented with G-418 (400 μg/μl) and of hygromycin (100 μg/μl). The medium for BJELM was supplemented with G-418 (400 μg/μl), hygromycin (100 μg/μl) and puromycin (0.5 μg/ml) and continuously grown with 10^-6^ M 4-hydroxytamoxyfen (4-OHT).

### TRAIL-induced apoptosis measurement

Cells were seeded in 24-well plates and incubated until the subconfluent state and incubated with recombinant human tumor necrosis factor-related apoptosis-inducing ligand (TRAIL; 200 ng/ml) for 8 h to monitor and measure apoptosis. The whole cell content, with floating (apoptotic) and attached cells, was collected for apoptosis measurement. Cell pellets were permeabilized on ice with 100 μg/ml digitonin and stained with APO2.7 (1:5; Beckman Coulter, USA). Apoptosis was measured by fluorescence-activated cell sorting (FACS) and quantified by detection of 7A6 mitochondrial antigen.

### Western blotting and antibodies

The whole cell protein extract was prepared using lysis buffer comprising 0.5 M LSBD (0.5 M NaCl, 50 mM Tris–HCl pH 7.9, 20 % glycerol, 1 % NP-40, 1 mM DTT), 0.3 % NP-40, 1× Protease Inhibitor Cocktail (Roche), 1 mM NaF, 10 mM Na_3_VO_4_, 1 mM PMSF, 0.125 μM okadaic acid. The protein concentration of extracts was measured using a Protein Assay reagent (Bio-Rad Laboratories). Proteins (50 μg) were separated by SDS PAGE, transferred to nitrocellulose membranes, and incubated with indicated antibodies. Antibodies used were as follows: c-MYC (N-262, rabbit; Santa Cruz, sc-764), SV40 T (Pab 108, mouse; Santa Cruz, sc-148), and β-actin (C-11, goat; Santa Cruz, sc-1615).

### Double nickase transfection by CRISPR-Cas9

Transfections were performed using double nickase plasmids using the manufacturer’s protocol and targeting the following factors: DHX33 (sc-404530-NIC2), CHD7 (sc-404017-NIC2), NOLC1 (sc-402907-NIC2), GTF3C4 (sc-411269-NIC2), PRMT3 (sc-406688-NIC2). Lipofectamin 2000 was used as the transfection reagent at a final concentration of 50 nM.

In brief, cells were seeded in six-well plates and grown for 24 h in antibiotic-free standard growth medium to achieve 80 % confluence. Transfection was performed with 1 μg of CRISPR plasmids followed by 24-h incubation. At the end of the incubation period the medium was replaced with a standard medium with antibiotics. Successfully transfected cells were sorted 24 h later by FACS, using green fluorescent protein as a marker, and used for other experiments.

### Test for anchorage-independent colony formation on soft agar

First, six-well plates were covered with “bottom agar” consisting of 4 % FCS, DMEM, and 0.7 % agar. Afterwards, 1000 cells (per one replicate) were mixed with a “top agarose” preparation consisting of DMEM 1×, 10 % FCS, and 0.35 % agar. The final mix was put on the top of the “bottom agar”. Cells were cultured with appropriate controls in soft agar medium for 21 days. Cells were fed once or twice per week with cell culture medium. Following this incubation period, formed colonies were stained with 0.5 ml of 0.005 % Crystal Violet for several hours and the number of colonies formed per well was quantified.

### Real-time quantitative PCR

Total RNA was extracted from cells using the GenElute™ Mammalian Total RNA Miniprep kit (Sigma). The extracted RNA (2 μg) was used for reverse transcription (AMV-RTase, Roche; Oligo(dT) New England Biolabs; 1 h incubation at 42 °C followed by 10 min at 94 °C). Transcribed cDNA was diluted tenfold and used for real-time quantitative PCR (RT-qPCR; Roche instrument LC480). For confirmation of introduced gene and marker gene expression the following primers were used: *TERT*, forward GCCTTCAAGAGCCACGTC, reverse CCACGAACTGTCGCATGT; *MYC*, forward CACCAGCAGCGACTCTG, reverse GATCCAGACTCTGACCTTTTGC; *CCND2*, forward GGACATCCAACCCTACATG, reverse CGCACTTCTGTTCCTCACAG; *THBS1*, forward CAATGCCACAGTTCCTGATG, reverse TGGAGACCAGCCATCGTC; *CHD7*, forward CACCTGAAGCATCACTGTAACAA, reverse TCACTTCTTGTCTTAGGTAGTACAGCA; *DHX33*, forward TGGTGAAAGCTGCACAGAAG, reverse CCATCGTAGCTGACATCACAA; *NOLC1*, forward ATAAGTTCGCCAAAGCGACA; reverse CTAAGAGGGAAGAGGCATTGG; *PRMT3*, forward AGGATGAGGACGATGCAGAT, reverse TTCTTCAGCAGATGTGAATAACCT; *GTF3C4*, forward TTGCTCCATGACAGCATTG; reverse GGGGCTTTGCAGTAACCTCT.

To assess relative gene expression, all qPCR measurements were normalized relative to the constitutively expressed GAPDH mRNA levels assessed with the following primers: *GAPDH*, forward CGACCACTTTGTCAAGCTCA; reverse AGGGGTCTACATGGCAACTG.

### Transcriptomics

Transcriptome analysis was performed using an Affymetrix Gene 1.0 ST Array in two biological replicates for each cell line, providing 1 μg of extracted RNA for library production. For comparing BJ, BJEL, and BJELM cells’ generated transcriptomes, we normalized all raw CEL files with the Affymetrix software Expression Console version 1.3.1 to calculate probe-set signal intensities using RMA algorithms with default settings. High reproducibility between the corresponding biological replicates was evaluated by calculating the Pearson correlation coefficient and skewness parameter between replicates and between BJEL and BJELM relative to BJ (Additional file 2: Figure S2).

To identify differentially expressed genes (DEGs), we compared BJEL versus BJ and BJELM versus BJ (in biological replicates). Thus, to identify confident DEGs, we used a modified *t*-test [6] for measurements coming from independent normal populations with unequal variances; this method aims to specifically address the question of differential expression in tests involving two samples (BJ versus BJEL or BJ versus BJELM) in which the experiments were performed in repeats. Finally, the probability of having a t-statistic value by chance was calculated and a threshold (significance level of 0.05) was applied.

### Inferring transcription factors involved in deregulated gene expression during cell transformation

**Fig. 2:**
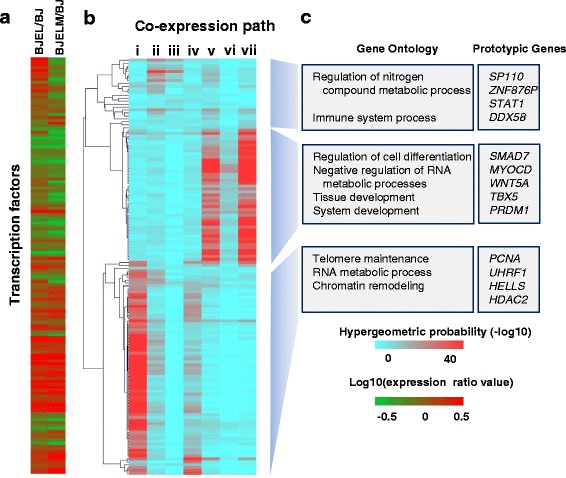
Association of key transcription factors (TFs) with co-expression pathways. Using the CellNet database of TF–TG associations revealed 142 TFs that were associated with more than 10 % of DEGs. **a** Expression ratios (relative to BJ) of TFs associated with particular co-expression pathways. **b** Heat map of hierarchical clustering illustrates the prevalence of corresponding TFs in the regulation of particular co-expression pathways (the *color bar* corresponds to the − log10(hypergeometric distribution value); *red* corresponds to high-confidence TF–TG associations, *blue* to low-confidence associations). **c** Biological process-based Gene Ontology analysis of clustered groups of TFs associated with particular co-expression pathways (*p* < 0.05) and prototypic genes

For the selection of transcription factors (TFs) associated with particular co-expression pathways we used the CellNet database of TF–target gene (TG) associations. We first selected TFs that are associated with >10 % of DEGs that constitute a given co-expression pathway. Then we selected TFs with promoter-associated RNA polymerase II (RNA Pol II), which gave rise to 142 TFs. Finally, we assessed the relevance of these TFs in distinct co-expression pathways using a hypergeometric distribution test with subsequent hierarchical clustering (Fig. 2).

### Chromatin immunoprecipitation assays

BJ, BJEL, and BJELM cells were fixed with 1 % paraformaldehyde (Electron Microscopy Sciences) for 10 min at room temperature. Chromatin immunoprecipitation (ChIP) assays were performed according to the following conditions: chromatin sonication and immunoprecipitation in lysis buffer (50 mM Tris–HCl pH 8, 140 mM NaCl, 1 mM EDTA, 1 % Triton, 0.1 % Na-deoxycholate) complemented with protease inhibitor cocktail (Roche 11873580001); two washes with lysis buffer; two washes with lysis buffer containing 500 mM NaCl; two washes with washing buffer (10 mM Tris–HCl pH 8, 250 mM LiCl, 0.5 % NP-40, 1 mM EDTA, 0.5 % Na-deoxycholate); two washes with TE buffer; elution at 65 °C; 15 min at 65 °C in elution buffer (50 mM Tris–HCl pH 8, 10 mM EDTA, 1 % SDS).

RNA Pol II (Santa Cruz sc-9001 H-224), H3K27me3 (Millipore 07-449), H3K4me3 (Abcam 8580), H3K9ac (Abcam 4441), and H3K27ac (Abcam 4729) antibodies were purchased from their corresponding commercial suppliers. RNA Pol II ChIP assays were performed with 3 × 10^6^ cells, while histone modification marks were evaluated with 1 × 10^6^ cells. All ChIP assays were validated using positive and negative controls. Specifically, enrichment performance was assessed at promoter regions of genes *SRSF6* and *NEK1* (for H3K4me3, H3K9ac, H3K27ac, RNA Pol II), *NEUROG1* and *MB* (for H3K27me3 validation), and *DPP10* as a cold region, using the following primers: *SRSF6*, forward CGTTCGACAACCAGCCCTT, reverse GGCCCGACTCACCCATTTT; *NEK1*, forward CGTTACCGCCTCTCCAACTT, reverse CTTACCCTACCCTGGCCTCT; *NEUROG1*, forward ACAGATAGAAAGGCGCTCAGA, reverse CGCAACTGGCACAGAGTAAC; *MB*, forward GGCTCACTGGGTGTCCTG, reverse AAGGTATAAAAACGCCCTTGG; *DPP10*, forward GTTTCCAATTTCATCCATGTCC, reverse CACATCAAACTGGTGGGTGA.

ChIP validation assays were performed by RT-qPCR (Roche instrument LC480 light cycler) using a QuantiTect kit (Qiagen).

### Massive parallel sequencing

qPCR-qualified ChIP assays were quantified (Qubit dsDNA HS assay kit, Invitrogen); 3–10 ng of the ChIP material was used for preparing Illumina sequencing libraries following a multiplexing approach (NEXTflexTM ChIP-seq Bioo Scientific, reference 5143-02). Prepared sequencing libraries were sequenced on an Illumina HiSeq2000 instrument. Regular Illumina pipelines were used for image processing and base calling. Sequence files were then aligned to the human genome assembly using default parameters (hg19; Bowtie).

### Quality control of sequence data

Sequence-aligned files were then qualified for enrichment performance using the NGS-QC Generator tool [7] (http://www.ngs-qc.org/). This methodology provides enrichment quality descriptors in a scale ranging from triple A (best) to triple D (worst). Based on this quantitative method, all ChIP-seq datasets described in this study had at least a triple B quality grade to ensure that only high quality datasets were used for downstream integrative analyses.

### Enrichment pattern detection and normalization of ChIP-seq intensity profiles

Relevant binding sites in all ChIP-Seq (except the H3K27me3 dataset, which was analyzed with the SICER tool [8]) datasets were identified with MeDiChI-Seq [9]. To enable a comparison of ChIP-seq profiles of the same target between different cell lines, a normalization procedure over profile global amplitudes prior to further analyses was applied using a Quintile-based approach. Briefly, we calculate read count intensity for a non-overlapping window of 100 bp across the genome and then normalize these intensities using quantile normalization from the “limma” package. Quantile normalization is a ranking-based approach where calculated read count intensities are sorted and ranked for each sample. The corresponding ranked values between samples are adjusted into a mean value. The impact of normalization was assessed using MA plots before and after normalization. First, we normalize all datasets associated with a given target; then normalized target datasets are brought to the same scale via a z-score normalization. A detailed description of this quantile normalization procedure, which is applicable for a variety of ChIP-seq and enrichment-related next-generation sequencing datasets and is available as part of Epimetheus, a user-friendly dedicated tool, is going to be presented in a further publication (in preparation).

### Integration of transcriptome and epigenome data

**Fig. 3:**
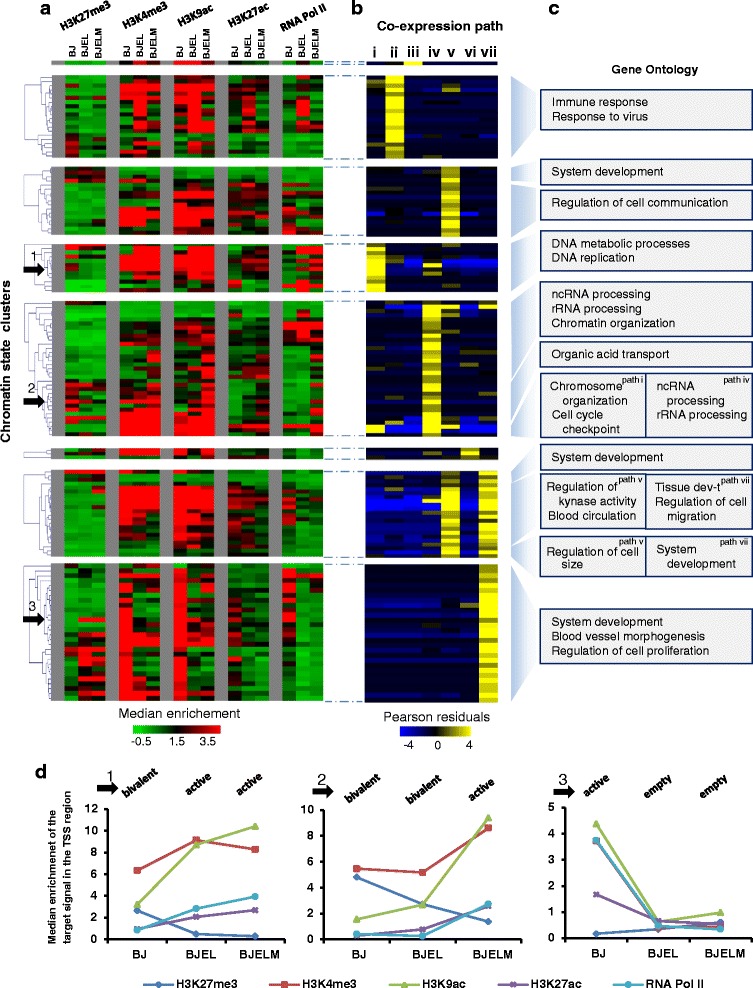
Chromatin state transitions in promoters of differentially expressed genes during the cell transformation process and integration of epigenetic data (chromatin state clusters) with transcriptome dynamics (co-expression pathways). **a** Hierarchical clustering of transcripts based on enrichment of histone modifications and RNA Pol II at the promoter of DEGs. The *color* represents the median enrichment for each cluster of genes within ±1.5 kb of a TSS of a DEG. **b** Heat map illustrating the prevalence of chromatin state clusters in particular co-expression paths. The *color* represents Pearson residuals. *Yellow* indicates significant enrichment of transcripts in the corresponding expression pathways with a corresponding chromatin state cluster. **c** Biological process-based Gene Ontology analysis of chromatin state clusters, regrouped by hierarchical clustering (hierarchical tree in **a**), and associated with the same co-expression pathway. **d** Three examples of chromatin state clusters illustrating the evolution of the epigenetic landscape in the stepwise transformation process (*black arrows* in **a**). *Panel 1* correspond to the changes from the bivalent chromatin state in BJ cells to the active state in BJEL and BJELM cells. In the same manner, *panel 2* corresponds to the changes from the bivalent chromatin state in BJ and BJEL cells to the active state in BJELM cells. Finally, *panel 3* corresponds to the chromatin state cluster that characterizes the group of downregulated genes in BJEL and BJELM cells; the promoters of these genes are in the active state in BJ cells but lose all marks in the BJEL and BJELM cells

To integrate transcriptome data with chromatin state dynamics, we performed unsupervised clustering of ChIP signals for each target that was assessed in the current study within a 1-kb window of each transcription start site (TSS) for all DEGs, comprising a total of 7616 transcripts. Histone marks or RNA Pol II binding were tagged as “present” at the TSS of the DEG if it satisfied the following criteria: (i) the peak was detected—the summit of the detected peak (by MeDiChI-Seq [9] or by SICER [8]) was 500 bp up- or downstream of the TSS of the DEG; (ii) the peak was of high intensity after normalization—following quantile and Z normalization the Z-score of a given peak was >1.65 (P_95_); (iii) the peak was robust—the signal had to be robust with less than 15 % dispersion after the subsampling procedure (NGS-QC tool, http://www.ngs-qc.org/). Afterwards, unsupervised clustering of all the possible combinations of histone marks and RNA Pol II at the TSS of DEGs was performed. A heatmap of chromatin state dynamics represents the median enrichment for each cluster of genes within ±1.5 kb of a TSS of a DEG at each stage of the stepwise transformation model (Fig. 3a, d). At the next step we assessed whether dynamic patterns of chromatin states are associated with particular groups of co-expressed genes (Fig. 3b, d).

### Gene regulatory network reconstruction

**Fig. 4:**
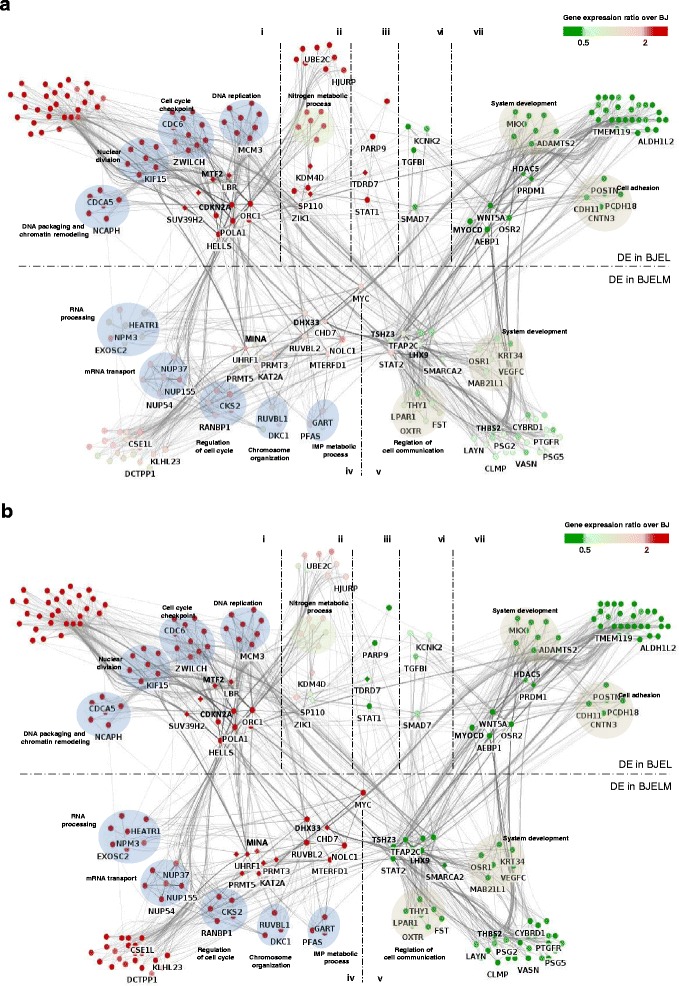
Gene regulatory network (GRN) of the BJ stepwise transformation system. **a** GRN of immortalized BJEL cells. **b** GRN of transformed BJELM cells. Chromatin remodelers/modulators are represented as *diamond-shaped nodes*, while other genes, highly connected “hubs”, and “bottlenecks” are represented as *circles*. The differential expression levels at immortalization and during the transformation steps were colored per node in a heat map format such that the dynamic changes could be visualized. *Dashed lines* separate the GRN into seven segments corresponding to seven (i to vii) gene co-expression pathways. Functionally related genes are *circled* under an enriched GO term (DAVID, *p* < 0.05)

To provide a comparative view of the signal transduction events governing the cell transformation in the stepwise model system, we reconstructed a gene regulatory network (GRN) by combining several layers of information from three different databases: (i) the MiMI, which contains protein–protein, DNA–protein, and other interaction data, querying the interactions only between DEGs [10]; (ii) CellNet, a collection of directed TF–TG interactions [11, 12], where the TFs listed in the CellNet Gene Regulatory Network (GRN) collection were associated with genes differentially expressed in the BJ/BJEL/BJELM model system; (iii) several publically available MYC-targeted ChIP-seq datasets (see “Methods”). The integration of DEG-related interactions in the Cytoscape platform (version 2.8.3) resulted in a dense cell type-specific GRN composed of 1265 nodes and 5327 edges which were then organized according to the transformation steps and gene co-expression pathways. Furthermore, a two-step GRN reduction process was applied by using a double screening system in the Hubba tool [13] to define the highly connected nodes (“hubs”). In addition, a second layer of topological metrics reduction was applied by scoring for “bottleneck” nodes since previous reports demonstrated that, in addition to highly connected nodes (“hubs”), bottleneck nodes (defined as those interconnecting highly connected nodes or hubs in the system) might represent highly relevant components in the system [14]. In particular, bottleneck nodes in signal transduction systems might correspond to essential entities required for the flow of the signal transduction driving the phenomenon of interest. Definition of hubs and bottlenecks was performed using topological metrics, such as MNC (Maximum Neighborhood Component), DMNC (Density of Maximum Neighborhood Component) and Bottleneck [13]. This reduction process generated GRNs composed of 253 nodes and 2657 edges. The organization of the reduced GRN and its visualization were performed with the Cytoscape package Cerebral [15]. As part of the visualization options in Cytoscape, the differential expression levels in BJEL and BJELM cells per node were presented in a heat map format such that the transcriptome dynamic changes could be visualized. The changes of node color for groups ii, iii, iv, and v in Fig. 4a and 4b indicate the change in expression of co-regulated genes during the transformation process.

### Analysis of publically available ChIP-seq datasets used for targeting MYC enrichment

The following ChIP-seq datasets from the Gene Expression Omnibus (GEO) were used to identify MYC enrichment sites: GSM1088663 (HeLa cells), GSM896988 and GSM1000576 (BJ cells), and GSM748557 (NHEK cells). The raw sequencing files were aligned with Bowtie using default parameters and processed with MACS for peak annotation. A threshold of − log10(*p* value) ≥300 was applied to select peaks with high confidence.

## Results

### Transcriptome dynamics during the cell transformation process

Following validation of the stepwise tumorigenesis model, which included the determination of TRAIL (tumor necrosis factor-related apoptosis-inducing ligand) sensitivity [16] (Additional file 2: Figure S2), we assessed the global gene expression in all three cell lines and the ratio of expression levels of immortalized to normal cells (BJEL/BJ) and cancer relative to normal cells (BJELM/BJ). Genes exhibiting >2- and <0.5-fold expression changes with a significance level of *p* < 0.05 (modified *t*-test, “Methods”) were considered up- and down-regulated, respectively, and classified as differentially expressed genes (DEGs). The resulting 1700 DEGs were subdivided into seven groups of co-regulated genes according to their expression characteristics during the subsequent steps of transformation (Fig. 1; Additional file 2: Figure S2a, b). Nearly half of the genes (47 %) showed an altered expression level at the pre-transformation step and 65 % of genes changed expression level after full transformation by c-*MYC* expression. Interestingly, about 12 % of these genes changed expression after both immortalization and c-MYC-mediated transformation.

Gene Ontology (GO) analysis revealed that each pathway of DEGs is enriched for distinct GO terms (Fig. 1b; Additional file 3: Figure S3a). Notably, those enriched in the group of genes that are down-regulated in transformed cells (pathway v) are associated with regulation of cell motion, cell communication, and regulation of cell differentiation, as well as suppression of angiogenesis, while genes that are progressively induced from the normal to the tumorigenic stage (pathway iv) are significantly enriched for the GO terms ribosome biogenesis and noncoding RNA and rRNA processing. Disease-related GO analysis using DAVID [17, 18] showed significant enrichment of DEGs characteristic for several types of cancers, among them breast, bladder, stomach, and lung cancer (Additional file 3: Figure S3b).

Together these results show that the stepwise transformation model shares multiple similarities with different types of human cancers and is a convenient and reliable cell model for tumorigenesis research. Importantly, several of the deregulated gene expression pathways affect phenomena that are well-described as hallmarks of cancer, such as the activation of angiogenesis, the activation of invasion and metastasis, and regulation of cell cycling [1].

### Multiple chromatin remodelers/modulators are dysregulated during cell transformation

To monitor changes of the epigenome during the stepwise BJ transformation process, we assessed first whether chromatin remodelers/modulators (CRMs) were deregulated. Indeed, we detected 24 differentially expressed CRMs, belonging to all three classes of writers, erasers and readers, and other chromatin remodelers (Additional file 4: Figure S4; Additional file 5: Table S1). Fourteen of these changed their expression at the last step of transformation as a consequence of the overexpression of c-*MYC*; interestingly, 12 genes among these were upregulated and are members of such functional groups as methyltransferases, acetyltransferases, demethylases, and related CRMs, indicating that MYC-induced transformation leads to dramatic changes in the epigenome involving CRMs.

The majority of CRMs defined in the current study are deregulated in different types of cancer, such as ovarian, bladder, lung, and many other types (see Additional file 5: Table S1 and Additional file 6 for references). For several CRMs, such as PRMT5 and MINA, the interaction with MYC was reported to be an essential step in cancer development (Additional file 5: Table S1, references 31, 32, 36–39 (listed in Additional file 6)). These observations suggest that CRMs are involved in regulation of tumorigenesis and mediate at least some of the transforming activities of overexpressed *c-MYC*. We would like to emphasize, moreover, that our present approach has identified new candidate CRMs, some of which are putative MYC targets that have not been previously recognized, two “writers” (GTF3C4 and PRMT3), three “readers” (LBR, AKAP1, and MBD5), and the PcG group member MTF2. *LBR* and *MTF2* are upregulated during the first step of transformation, while the other CRMs are deregulated upon c-*MYC* overexpression. Inspection of the publically available MYC cistrome of HeLa cells [19] revealed the presence of high-confidence (see “Methods”) MYC-binding sites in the *PRMT3* and *GTF3C4* promoters. Considering that these two genes are induced after MYC overexpression, *GTF3C4* and *PRMT3* are most probably direct targets of MYC in the BJ system.

We conclude from these results that: (i) LBR and MTF2, both involved in transcription repression (Additional file 5: Table S1), are potential regulators of the immortalization process and/or cooperate with the oncogene in the second step; and (ii) PRMT3, GTF3C4, AKAP1, MBD5, and GTF3C4 are new players in the tumorigenesis process which mediate the MYC-dependent effect on chromatin remodeling.

### In silico prediction of key TFs involved in deregulated gene expression during cell transformation

To reconstruct the alterations in the activity of TFs during the steps that lead to cell transformation in the BJ model, we integrated information on TF–TG associations described in the CellNet database [11, 20]. This led to the identification of 142 TFs (Additional file 7: Table S2), of which 42 are differentially expressed during stepwise tumorigenesis (Fig. 2a). Sorting these TFs for their association with particular co-expression pathways led to a clustering of groups of TFs that were preferentially associated with one or more co-expression pathways (Fig. 2b). According to the hierarchical clustering of TF-specific association with co-expression pathways, we could distinguish at least three subgroups: TFs that are preferentially associated with pathways i and iv, with pathways ii and iii, or with pathways v and vii. Importantly, these distinct groups of co-expression pathway-associated TFs are apparently involved in regulating specific cell biological functions, as revealed by the corresponding GO analysis (Fig. 2c). Specifically, TFs associated with pathways v and vii are involved in regulation of cell differentiation and tissue development, while co-expression pathway i-associated TFs are primarily involved in telomere maintenance and chromatin remodeling.

Notably, co-expression pathway ii comprises genes involved in immune and defense responses; STAT1 is among the TFs that are specifically associated with these co-regulated genes. That *STAT1* is upregulated in pre-transformed cells may reflect the established role of this factor in cell autonomous anti-tumor immune response [21] (Fig. 1; Additional file 3: Figure S3; pathway ii). In addition, STAT1 is known to negatively regulate angiogenesis, tumorigenicity, and metastasis of tumor cells [22] and suppresses mouse mammary gland tumorigenesis [23]. Downregulation of *STAT1* by c-*MYC* overexpression observed in the current study is also detected in Burkitt’s lymphoma [24], supporting the concept that immune escape of tumor cells could be promoted by activation of a cellular oncogene [24].

Several functionally related (GO term 45595: regulation of cell differentiation) TFs, such as MYOCD, TWIST1, TBX5, and SMAD7, which are known to be involved in cancer development and/or sustainment [25–27], are specifically associated regulators of genes that are down-regulated along the cell transformation process in our model system (pathways v, vi, and vii). In particular, myocardin (MYOCD), a transcriptional co-factor for smooth muscle cell-specific genes that has been shown to block human mesenchymal transformation [28], was down-regulated in pre-transformed cells. Thus, decreased *MYOCD* expression may contribute to an increased proliferative potential of pre-transformed and transformed cells. In addition, it is likely to contribute to the concomitant loss of fibroblast identity and gain of stem cell identity as revealed by cell identity analysis using CellNet [11] (Additional file 8: Figure S5).

Another functionally related group of TFs that are associated with co-expression pathways i and iv are involved in chromatin remodeling. Among these are UHRF1, HELLS, and HDAC2, all of which are known to affect the tumorigenesis process [29, 30]. Remarkably, RUVBL2/TIP49, a member of the same group that is upregulated in BJELM cells and is known to interact with c-MYC, has been reported to regulate β-catenin-mediated neoplastic transformation [31].

Altogether, the observed associations reveal that the stepwise tumorigenesis model recapitulates aberrations of several regulatory systems, ranging from cell autonomous immune responses to chromatin remodeling and cell (de)differentiation, all of which are features previously reported to be altered in human cancer.

### Cell transformation significantly impacts on chromatin state dynamics

Given the known deregulation of cancer epigenomes due to mis-expression or mutation of epigenetic factors [32, 33], the de-regulation of CRMs in the BJ system, and the fact that c-MYC recruits a variety of epigenetic factors and chromatin remodelers to its targets [34, 35], we performed a genome-wide analysis of chromatin state transitions for all three steps of the cell transformation. We used chromatin immunoprecipitation (ChIP) coupled with massive parallel sequencing (ChIP-seq) for several functionally interpretable histone modifications, including H3K27me3 (inactive promoters), H3K4me3, H3K9ac (active promoters), and H3K27ac (active promoters and enhancers). We also determined the chromatin association of RNA Pol II, which is generally enriched at the transcriptional start sites (TSSs) of active promoters.

In view of the dynamic nature of gene expression observed during the tumorigenesis process, we focused on elucidating histone modification patterns at TSSs. To identify gene promoters with a similar pattern, we performed unsupervised clustering of all the possible combinations of histone marks and RNA Pol II-normalized ChIP signals (see “Methods”; Additional file 9: Figure S6) within 1.5 kb up- and downstream of each TSS for all DEGs, comprising a total of 7616 transcripts (Additional file 10). We detected 26 different combinations of histone marks at DEG promoters and classified them into seven chromatin states (Additional file 11: Figure S7): (a) active (RNA Pol II and at least two histone marks of active transcription are present); (b) weakly active (RNA Pol II and only one histone mark of active transcription); (c) transcription-prone (at least two histone marks of active transcription but no RNA Pol II); (d) bivalent (any of states a to c but accompanied by repressive H3K27me3 marks); (e) ambiguous (only one histone mark or RNA Pol II alone); (f) empty (no signal); and (g) repressed (only H3K27me3). Further, the dynamic changes in chromatin states at the promoters of DEGs through the stepwise transformation process and all possible combinations of chromatin state evolution (chromatin state transitions) were assessed, giving rise to 135 chromatin state clusters, and integrated with the transcriptome changes along the transformation process (Fig. 3).

The majority of clusters revealed highly dynamic chromatin patterns, suggesting that chromatin state regulation of DEGs is tightly linked to, and possibly controls to a significant degree, DEG expression and, thus, the acquisition of the pre-transformed and transformed cell states. The differential regulation of CRMs indicates a tight linkage between, and mutual regulation of, DEGs (including TFs) and CRMs. Interestingly, genes sharing the same co-expression pathway could be further subdivided according to their chromatin state transitions into groups of genes with related functionalities. For example, co-expression pathway iv comprises genes overexpressed at the second step of transformation associated with chromatin patterns, such as gain in activating H3K4me3 and H3K9ac marks in the absence of repressive H3K27me3; this group of genes is involved in rRNA and noncoding RNA processing and chromatin organization. In contrast, a second group sharing the same co-expression pathway, which loses H3K27me3 with a concomitant gain of H3K4me3 and H3K9ac marks, is predominantly linked to organic acid transport (Fig. 3c). Thus, groups of functionally related genes can be distinguished at the chromatin level despite their similar expression patterns.

### Reconstruction of GRNs

To provide a comprehensive view of the signal transduction events governing the cell transformation in the stepwise human cellular tumorigenesis model, we reconstructed a GRN integrating gene interaction data from publically available databases with gene expression data from our experiments (see “Methods” for details). This integration process resulted in the establishment of a comprehensive GRN of 1265 nodes and 5327 edges.

To explore the functionally most relevant aspects of the reconstructed network, we reduced its complexity by applying topological criteria to identify highly connected (“hubs”) and key connector nodes (“bottlenecks”) that are relevant to the investigated signal transduction processes [14]. The reduced network of 253 nodes and 2657 edges (Fig. 4a, b) shows the connectivity between the major nodes, which are possibly functionally involved in the cell transformation process. The network is divided into two parts, showing key regulatory nodes differentially expressed on the first step of transformation in the upper part, while those changing expression levels after c-*MYC* overexpression are depicted in the lower part. Dashed lines in Fig. 4 split the network landscape into seven sections to place co-expressed genes in proximity to each other. The flow of signal goes from the BJEL state (upper part) through the MYC to other TFs and TGs in the BJELM state (lower part). In addition, functionally related highly connected nodes are grouped together in the context of the corresponding enriched GO terms to reveal subprograms, such as regulation of cell adhesion, cell communication, or RNA processing, all of which are hallmarks of cell transformation. In the centre of the network we placed the bottleneck genes, which are supposed to direct the flow of signaling information from the functionally related hubs to the target genes (not shown in the reduced network). The reconstructed gene network pointed towards bottleneck genes that are key factors, like the RNA biogenesis-linked *NOLC1*, *DHX33*, and *CHD7*, as potential key regulators of cell transformation and direct downstream targets of c-MYC, based on the ChIP-seq analysis of publically available data sets (“Methods”, Additional file 12). These genes are pivotal for RNA metabolic processes [36–38]. Interestingly, previous studies have shown a correlation between the upregulation of these genes and tumor progression and, indeed, marked increases in rRNA synthesis is a general attribute of many types of cancers [39, 40], suggesting that changes in rRNA synthesis may be a prerequisite alteration in cell transformation. DHX33 has been reported to be an important mediator of rRNA synthesis and cell growth [41]. Furthermore, following the fact that rDNA transcription is greatly influenced by the *RAS*, *MYC*, and *NPM* oncogenes, *DHX33* upregulation was shown to be required for enhanced transcription during RAS activation and for RAS-initiated tumor progression [37]. The observations that *DHX33* is overexpressed in our system following *cMYC* overexpression and has a MYC binding site in its promoter (GSM1088663) suggest that DHX33 is a mediator of MYC signaling. Other key factors that became apparent from these GRNs include TSHZ3, previously reported to correspond to a novel potential tumor suppressor [42], and LHX9, which is aberrantly methylated and downregulated in malignant gliomas of childhood [43]. Thus, the present reconstructed GRN is a rich source of (novel) regulators of tumorigenesis that could be further studied in suitable (in vivo) systems.

### Validation of predicted factors

**Fig. 5:**
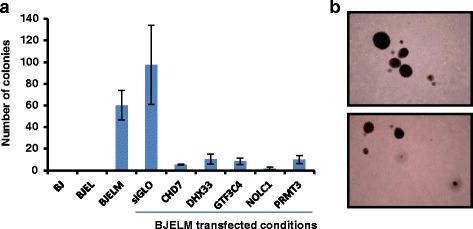
Validation of predicted factors. **a** Test for anchorage-independent growth on soft agar. All BJELM transfected conditions, except for the control, exhibit drastic decreases in the capacity to form colonies on soft agar. **b** Colonies formed by BJELM cells after 3 weeks of incubation on soft agar. The error bars represent the standard deviation between the biological replicates

With the aim of evaluating the biological relevance of the reconstructed GRNs, we assessed the role of the TFs DHX33, NOLC1, and CHD7 as well as that of the CRMs PRMT3 and GTF3C4, all of which act “downstream” of MYC, in cell transformation. Specifically, we used the CRISPR-Cas9 technology to inactivate these genes in BJELM cells and evaluated the consequence of this perturbation on the tumorigenic properties that had been acquired in these cells by the overexpression of c-*MYC* relative to the isogenic non-tumorigenic BJEL precursor cells. For this we used a well-established tumorigenesis assay, namely the acquisition of anchorage-independent growth on soft agar; this assay is widely used as a predictor of tumorigenicity and is considered one of the most stringent assays for studying the malignant transformation of cells [44]. In fact, as illustrated in Fig. 5a, normal BJ and immortalized BJEL cells are not able to grow in an anchorage-independent manner, while the overexpression of c-*MYC* conferred onto BJELM cancer cells the ability to proliferate under these conditions (Fig. 5a, b). Importantly, CRISPR-Cas9-mediated individual inactivation of all tested “downstream” factors of Myc (*CHD7*, *DHX33*, *GTF3C4*, *NOLC1*, and *PRMT3* genes) resulted in a drastic drop in the ability of BJELM cells to form colonies on soft agar, while BJELM cells, as well as mock-transfected BJELM cells (“siGLO”), showed efficient colony formation in soft agar (Fig. 5a). Together our data reveal that each of these factors plays a critical role in mediating key oncogenic effects of *MYC* overexpression in this isogenic model system.

## Discussion

### Isogenic cellular tumorigenesis models are versatile tools for systems biology studies

Any comparative analysis of normal and tumor cells with the aim of identifying the mutational and deregulatory events that cause cancer is seriously limited, and may even be impossible, if using established cell lines or patent-matched normal and tumor samples. Established cell lines have acquired extensive genetic alterations to support continuous growth in culture (“immortalization”) and to escape senescence and/or other failsafe programs [45]. In addition, cancer cells are genetically unstable and carry many genetic abnormalities accumulated due to various conditions, including infections during tissue culture. When using normal and tumor tissue sections from the same patient, the tumor history is generally unknown and both genome instability and clonal heterogeneity/selection limit any comparative data analysis. Yet, it is important to understand the deregulation which occurs at multiple gene regulatory levels when a cell converts into a tumor cell by a minimal set of genetic alterations. Moreover, cancer genomics provides us with sets of “driver” and “passenger” genes, whose implication, alone and in combination, in the tumorigenic process is only known for a very small subset. Thus, there is a need for a model system which can be engineered and recapitulates the basic features of a tumor cell. Such a system was originally developed by Hahn and Weinberg [3] and has been used in this study to decipher the regulatory levels and gene regulatory networks (GRNs) that are altered by “simple” engineered tumorigenesis of primary human cells.

This system is virtually isogenic, thus granting the possibility to dissect GRNs reflecting system deregulation due to the introduction of defined genetic elements. In the present system the overexpressed catalytic subunit of hTERT protects BJ cells from telomere erosion [3, 46]. In addition, large T and small t-antigen expressed from the SV40 early region inactivate the tumor suppressors RB and P53, thus allowing the cells to evade antiproliferative and apoptotic signals [47]. Finally, overexpression of c-*MYC*, often upregulated through either a stabilization mutation or gene amplification in a wide variety of human cancers, transforms the cells into bona fide cancer cells [48]. Though such a system may seem reduced and simple compared with tumorigenesis in the animal, it nevertheless enabled us to decipher the underlying deregulated gene networks, including alterations of TF activities, and to identify transformation-associated deregulation of epigenome modifiers. As could be expected, GO analysis of DEGs yielded GO terms indicative of cell transformation. Indeed, a marked increase in rRNA synthesis is a general attribute of many cancers [40, 41] and rRNA transcription was shown to be stimulated by c-MYC [49]. This correlates with our observations showing MYC-mediated upregulation of genes functionally related to RNA biogenesis, such as *DHX33*, *HEATR1*, *NOLC1*, and others (Fig. 4a, b, RNA processing functional group). Notably, disease-oriented functional annotation clustering showed that DEGs in the stepwise BJ transformation system comprise genes that are implicated in different types of cancer, such as breast or bladder cancer. In addition, we used cBioPortal [50, 51] to see if genes identified in this study can be correlated with publically available datasets of human cancers (cBioPortal is an exploratory analysis tool that, among others, hosts TCGA (The Cancer Genome Atlas) datasets ready for network analysis). From the cross-cancer alteration analysis under the simultaneous query of MYC, NOLC1, DHX33, and CHD7, a large number of cancers possess alterations in these genes (Additional file 13: Figure S8). In particular breast cancer, neuroendocrine prostate cancer, and ovarian serous cystadenocarcinoma have the highest rate of alteration of these genes in tumor samples (62.1, 53.3, and 45 % of cases, respectively), suggesting that our model recapitulates some traits of real tumor samples. This indicates that the BJ model can be used to determine key gene regulatory principles of the transformation process which can also be observed in “real” human tumors. Moreover, the availability of CRISPR technologies facilitates the engineering of such isogenic systems from primary human cells to model the process of tumorigenesis and assess the contribution of (combinations of) aberrations by introduction of genetic elements which are found deregulated or mutated in human tumors.

### Deregulation of CRMs and the epigenome landscape in tumorigenesis: mutual inter-relationship

Increasing evidence suggests that many epigenetic regulatory proteins are deregulated in cancer and that histone mark patterns are globally changed within the cancer epigenome [32, 52]. Our observations support this as a number of CRMs are differentially expressed during cell transformation, including the classes of “writers” and “erasers”. Most of them have been reported to play a role in tumorigenesis and their expression patterns in transformed BJELM cells are similar to those in several types of cancer (Additional file 4: Figure S4; Additional file 5: Table S1), indicating that the BJ stepwise transformation system is capable of recapitulating the deregulation of molecular pathways seen in “real” cancer and possibly can identify new regulators of tumorigenesis. In this respect, we point out several CRMs that have not been previously associated with tumorigenic cell transformation.

Deregulation of CRMs in the BJ model, which does not suffer from genome instability, reveals the epigenetic consequences of hTERT, SV-40 T and t antigen, and MYC introduction and, thus, the mutual interconnection between transcription factor deregulation and epigenome alteration on the pathway towards tumorigenesis. This would not be possible by comparing non-isogenic normal and cancer cell lines, as genome instability of cancer cells leads to merging of effects due to the introduction of exogenous elements and those coming from genome aberrations already existing in the tumor cell line.

### GRNs of tumorigenesis

Cellular phenotypes are determined by the temporal regulation and dynamics of networks of co-regulated genes. Thus, elucidating GRNs is crucial for understanding of normal and cancer cell functioning. Until today only a few studies have addressed this issue—for example, the elucidation of the P53 regulatory network [53] or the analysis of locus expression signatures from retroviral insertion-induced tumorigenesis [54]. To perform a systematic analysis of GRNs underlying tumorigenesis, we used a novel combinatorial approach by (i) integrating transcriptome data during transformation steps with chromatin state dynamics, (ii) complementing this with an analysis of CRMs involved in this process, and (iii) inferring key transformation-related TFs by using a database of established TF–TG associations from multiple human lineages. The reconstructed GRNs reveal a crosstalk between the elements perturbing the normal system through TFs and CRMs as “transformation mediators” to the executor nodes, thus giving a comprehensive view of the molecular chain of events. The present approach could be applied to dissecting other processes, like cell differentiation or cell fate reprogramming, and the decryption of cause-and-consequence mechanistic links.

## Conclusions

In the current study we reconstructed GRNs that are altered during the process of stepwise human cellular tumorigenesis, providing a rich source of (novel) regulators of tumorigenesis. Using the reconstructed network, we predict and validate several transcription factors as being key players in the establishment of tumorigenic traits of transformed cells. Our data reveal the importance of CRMs in oncogene-induced tumorigenesis and identify new CRMs involved in this process.

## Availability of data and materials

SNP arrays, Affymetrix microarrays and Illumina platform ChIP-seq data sets supporting the results of this article are available in the Gene Expression Omnibus repository under the accession number GSE72533 (http://www.ncbi.nlm.nih.gov/geo/query/acc.cgi?acc=GSE72533).

## Additional files

Additional file 1: Figure S1. SNP analysis. **a** Statistics of changes in single-nucleotide polymorphisms (*SNP*), loss of heterozygosity (*LOH*), and copy number (*CN*) in the BJ stepwise transformation system in comparison with a previously used MCF10A-derived breast cancer tumorigenesis system (*MCF10A*, non-tumorigenic breast cell line; *MCF10AT*, premalignant breast cell line generated by HRAS transformation of MCF10A; *MCF10CA1a*, poorly-differentiated malignant breast cell line derived from a MCF10AT xenograft). Note the nearly isogenic character of the BJ stepwise transformation system and the high genetic divergence in the MCF10A system. **b** Chromosome diagrams illustrating changes in copy number in immortalized BJEL and transformed BJELM cells relative to normal BJ cells. *Red* and *blue triangles* correspond to loss and gain in copy number, respectively. (PDF 2.96 mb) Additional file 2: Figure S2. Description and validation of the BJ stepwise tumorigenesis system. **a** The expression pattern of co-regulated genes based on comparative transcriptomics of primary, immortalized, and transformed cells. **b** Statistics of changes in differentially expressed genes during cell transformation. **c** RT-qPCR validation of exogenous (*cMYC*, *TERT*) and transformation-relevant (*CCND2*, *THBS1*) gene expression. **d** Western blot analysis of whole cell extracts of BJ, BJEL, and BJELM cells confirms overexpression of T antigen and MYC-ER during the immortalization and tumorigenic steps, respectively. **e** Validation of the determination of TRAIL sensitivity during the transformation process. TRAIL-induced apoptosis was observed specifically in BJELM cells, while BJ and BJEL cells showed resistance to TRAIL treatment. **f** Reproducibility between replicates evaluated by calculation of the Pearson correlation coefficient. **g** Reproducibility between replicates evaluated by calculation of the skewness parameter between BJEL and BJELM replicates relative to BJ replicates. (PDF 2.95 mb) Additional file 3: Figure S3. Gene Ontology (GO) analysis of differentially expressed genes in the stepwise tumorigenesis system. **a** Functional clustering by biological pathway using DAVID for each set of co-regulated genes (pathways i to vii). **b** Disease-related GO (DAVID) of differentially expressed genes. The *x-axis* (*p* value) is given as − log(*p* value). Illustrated GO terms have *p* value <0.05. (PDF 2.96 mb) Additional file 4: Figure S4. Gene expression levels of chromatin remodelers/modulators (CRMs) differentially expressed in the stepwise tumorigenesis system. CRMs are specified on the *left* together with the corresponding co-expression pathways. The heatmap shows the ratio of expression in BJEL or BJELM cells relative to the expression in BJ cells. The corresponding color code is shown on the *right*. (PDF 2.95 mb) Additional file 5: Table S1. Chromatin remodelers/modulators differentially expressed during cell transformation. The table describes their reported function and involvement in cancer and provides the corresponding references (listed in Additional file 6). Abbreviations: *BRD* bromodomain, *CHD* chromodomain, *HAT* histoneacetyltransferases, *HMT* histonemethyltransferase, *TDRD* Tudor domain. (PDF 563 kb) Additional file 6. Supplementary data references. (DOCX 21 kb) Additional file 7: Table S2. Transcription factors preferentially associated with specific co-expression pathways and which originate from deregulated gene programming during tumorigenesis. Some of these TFs are differentially expressed as well and the co-expression pathway they belong to is shown in the last column. (PDF 102 kb) Additional file 8: Figure S5. Classification heatmap model showing the loss of fibroblast identity by BJ fibroblasts during the transformation, while gaining traits of embryonic stem cells. The analysis was performed using the CellNet tool. The *color key* shows the similarity between the training system and study samples. *Yellow* and *black* indicate high and low levels of resemblance, respectively. *b.r.* biological replicate. (PDF 2.96 mb) Additional file 9: Figure S6. A two-step normalization procedure required for proper multiprofile comparison. **a** To account for technical aspects like antibody efficiency and sequencing depth, we used Epimetheus, a two-step normalization procedure in which (i) the raw count intensity in ChIP-seq datasets produced with antibodies targeting the same factor are corrected following a quantile normalization procedure; then (ii) normalized ChIP-seq profile read counts corresponding to a variety of factors are brought to the same scale via a z-score normalization correction. **b** The effect of the quantile normalization on H3K9ac datasets assessed in all three cell lines of the stepwise transformation model. Notice that BJELM cells display lower intensity levels of the H3K9ac mark in the *LBR* promoter (*blue arrow*) relative to BJ and BJEL cells, while *LBR* gene expression is upregulated in BJEL and BJELM cells; after quantile correction, the levels in the BJELM dataset appears the same as in the BJEL dataset, both higher than in the BJ dataset. Furthermore, notice the higher background (region under the *red brace*) in the raw profiles of the BJEL dataset (in comparison with BJ and BJELM), which is brought to the same level in all three datasets after normalization. (PDF 520 kb) Additional file 10. Transcriptome data summary provided in Excel format. (XLS 1293 kb) Additional file 11: Figure S7. Chromatin state annotation. **a** Statistical analysis of chromatin states (initial and combined) at the TSSs of DEGs. **b** Chromatin state classification. **c** Normalized ChIP signal intensities at the TSSs ±500 bp, ordered from first to 26th chromatin state as in **a** in BJ cells. (PDF 2.95 mb) Additional file 12. MYC target genes as determined by the analysis of publically available ChIP-seq profiles and defined by the association of the MACS peaks (*p* value threshold = −30) with the TSS of genes using a 10-kb distance as an association criterion. (XLS 199 kb) Additional file 13: Figure S8. Cross-cancer alteration summary for CHD7, DHX33, NOLC1, and MYC among 123 cancer types; 99 cancer types that have alterations in these genes are displayed in the histogram. In 49 types of cancer, alterations in these genes occur in more than 10 % of cases. In particular, breast cancer, neuroendocrine prostate cancer, and ovarian serous cystadenocarcinoma have the highest rate of amplification of these genes in tumor samples (55.2, 50.5, and 44.1 % of cases, respectively). (PDF 2990 kb)

## Abbreviations

ChIP: chromatin immunoprecipitation
CRM: chromatin remodeler/modulator
DMEM: Dulbecco’s modified Eagle’s medium
FCS: fetal calf serum
GO: Gene Ontology
GRN: gene regulatory network
RNA Pol II: RNA polymerase II
SNP: single-nucleotide polymorphism
TRAIL: tumor necrosis factor-related apoptosis-inducing ligand
TF: transcription factor
TF–TG: transcription factor–target gene
TSS: transcription start site
