# Supplementary material for: Reconstruction of gene regulatory networks reveals chromatin remodelers and key transcription factors in tumorigenesis
Source: Genome Med. 2016 May 19;8:57. doi: 10.1186/s13073-016-0310-3 (PMC4872343; doi:10.1186/s13073-016-0310-3)
Supplement: Additional file 1: Figure S1. — SNP analysis. a Statistics of changes in single-nucleotide polymorphisms (SNP), loss of heterozygosity (LOH), and copy number (CN) in the BJ stepwise transformation system in comparison with a previously used MCF10A-derived breast cancer tumorigenesis system (MCF10A, non-tumorigenic breast cell line; MCF10AT, premalignant breast cell line generated by HRAS transformation of MCF10A; MCF10CA1a, poorly-differentiated malignant breast cell line derived from a MCF10AT xenograft). Note the nearly isogenic character of the BJ stepwise transformation system and the high genetic divergence in the MCF10A system. b Chromosome diagrams illustrating changes in copy number in immortalized BJEL and transformed BJELM cells relative to normal BJ cells. Red and blue triangles correspond to loss and gain in copy number, respectively. (PDF 2.96 mb) [file 13073_2016_310_MOESM1_ESM.pdf]

A

	BJEL	BJELM	MCF10AT	MCF10CA1a
	Relative to BJ		Relative to MCF10A	
**LOH**	0,00%	0,00%	0,83%	15,67%
**CN**	0,04%	0,36%	7,76%	17,06%
**SNP**	0,20%	0,37%	0,29%	4,37%

B

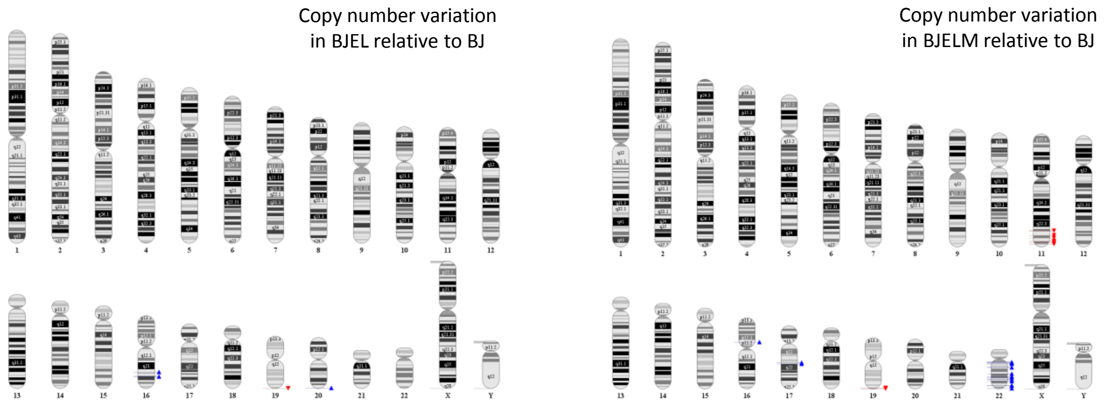

Figure S1. SNP analysis. a, Statistics of changes in Single-Nucleotide Polymorphism (SNP), Loss of Heterozygosity (LOH) and Copy Number (CN) in BJ stepwise transformation system in comparison to previously used MCF10A derived breast cancer tumorigenesis system (MCF10A – non-tumorigenic breast cell line; MCF10AT - premalignant breast cell line generated by HRAS transformation of MCF10A; MCF10CA1a poorly-differentiated malignant breast cell line derived from a MCF10AT xenograft[64,65]). Note the nearly isogenic character of BJ stepwise transformation system and the high genetic divergence in the MCF10A system. **b,** Chromosome diagrams illustrating changes in copy number in immortalized BJEL and transformed BJELM cells relative to normal BJ cells. Red and blue triangles corresponds to loss and gain in copy number, respectively.
