# Supplementary material for: Reconstruction of gene regulatory networks reveals chromatin remodelers and key transcription factors in tumorigenesis
Source: Genome Med. 2016 May 19;8:57. doi: 10.1186/s13073-016-0310-3 (PMC4872343; doi:10.1186/s13073-016-0310-3)
Supplement: Additional file 2: Figure S2. — Description and validation of the BJ stepwise tumorigenesis system. a The expression pattern of co-regulated genes based on comparative transcriptomics of primary, immortalized, and transformed cells. b Statistics of changes in differentially expressed genes during cell transformation. c RT-qPCR validation of exogenous (cMYC, TERT) and transformation-relevant (CCND2, THBS1) gene expression. d Western blot analysis of whole cell extracts of BJ, BJEL, and BJELM cells confirms overexpression of T antigen and MYC-ER during the immortalization and tumorigenic steps, respectively. e Validation of the determination of TRAIL sensitivity during the transformation process. TRAIL-induced apoptosis was observed specifically in BJELM cells, while BJ and BJEL cells showed resistance to TRAIL treatment. f Reproducibility between replicates evaluated by calculation of the Pearson correlation coefficient. g Reproducibility between replicates evaluated by calculation of the skewness parameter between BJEL and BJELM replicates relative to BJ replicates. (PDF 2.95 mb) [file 13073_2016_310_MOESM2_ESM.pdf]

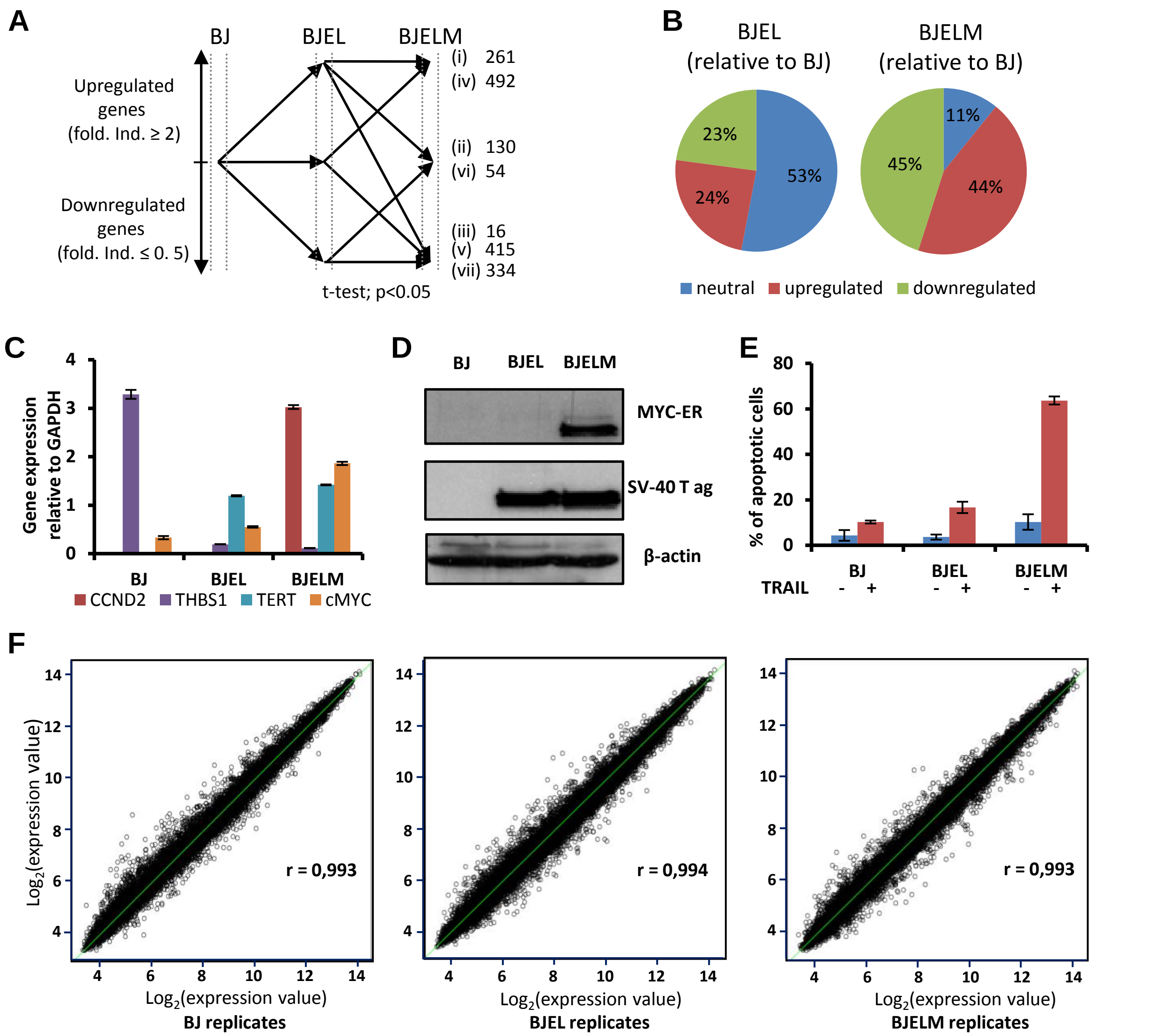

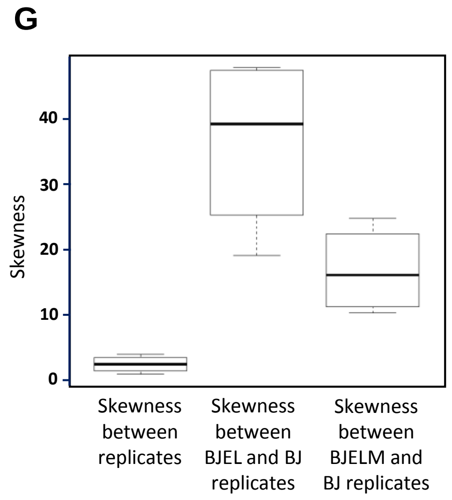

Figure S2. Description and validation of BJ stepwise tumorigenesis system. a, The scheme illustrates expression pattern of co-regulated genes based on comparative transcriptomics of primary, immortalized and transformed cells. **b,** Statistics of changes of differentially expressed genes during cell transformation. **c,** RT-qPCR validation of exogenous (*cMYC*, *TERT*) and transformation-relevant (*CCND2*, *THBS1*) gene expression. **d,** Western blot analysis of whole cell extracts of BJ, BJEL and BJELM cells confirms overexpression of T antigen and MYC-ER at the immortalization and tumorigenic steps, respectively. **e,** Validation of acquisition of TRAIL sensitivity[66] during the transformation process. TRAIL-induced apoptosis was observed specifically in BJELM cells, while BJ and BJEL cells are showing resistance to TRAIL treatment. **f,** Reproducibility between replicates evaluated by calculation of Pearson correlation coefficient **g,** Reproducibility between replicates evaluated by calculation of skewness parameter between BJEL and BJELM replicates relative to BJ replicates.
