# Supplementary material for: Reconstruction of gene regulatory networks reveals chromatin remodelers and key transcription factors in tumorigenesis
Source: Genome Med. 2016 May 19;8:57. doi: 10.1186/s13073-016-0310-3 (PMC4872343; doi:10.1186/s13073-016-0310-3)
Supplement: Additional file 3: Figure S3. — Gene Ontology (GO) analysis of differentially expressed genes in the stepwise tumorigenesis system. a Functional clustering by biological pathway using DAVID for each set of co-regulated genes (pathways i to vii). b Disease-related GO (DAVID) of differentially expressed genes. The x-axis (p value) is given as − log(p value). Illustrated GO terms have p value <0.05. (PDF 2.96 mb) [file 13073_2016_310_MOESM3_ESM.pdf]

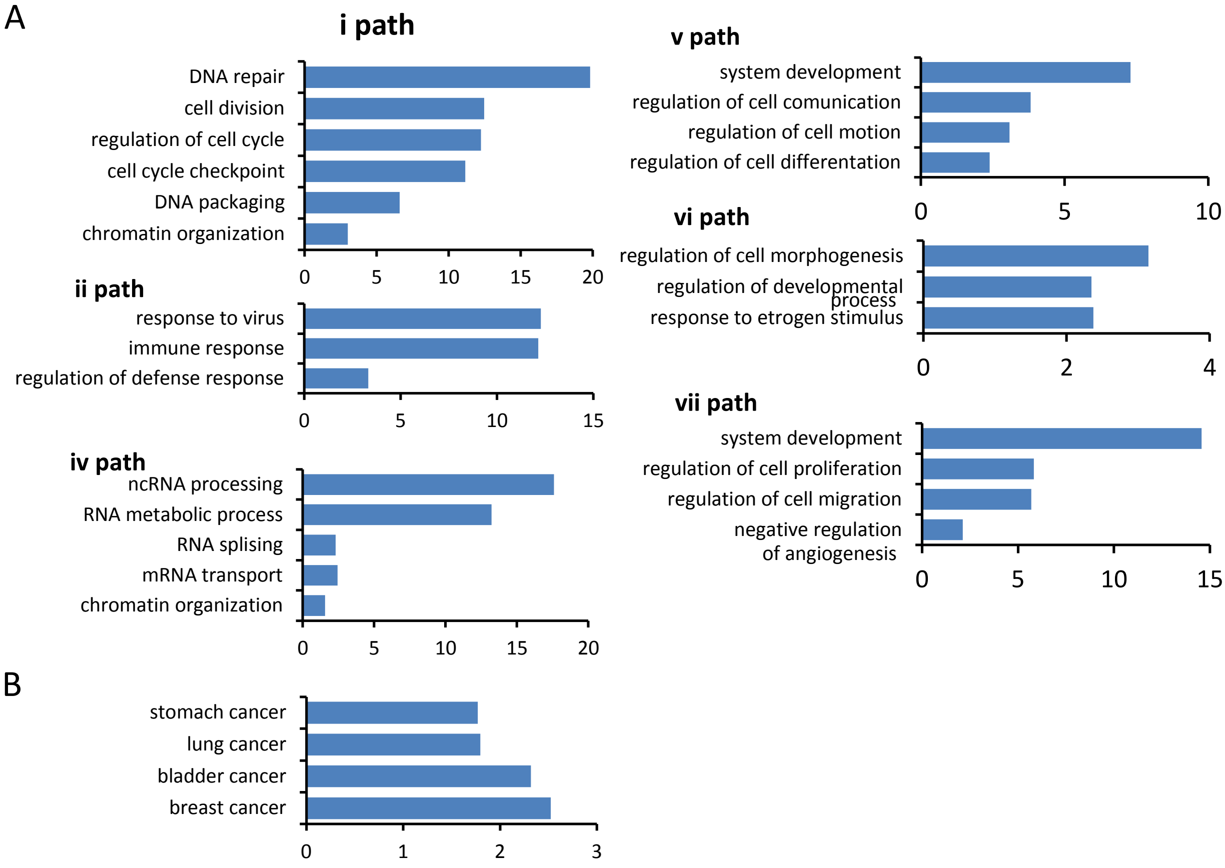


**Figure S3. Gene Ontology (GO) analysis of differentially expressed genes in stepwise tumorigenesis system. a,** Functional clustering by biological path using DAVID for each set of co-regulated genes (paths i to vii). **b,** Disease-related GO (DAVID) of differentially expressed genes. The x-axis (p-value) is given as –log(p-value). Illustrated GO terms have p-value < 0,05.
