# Supplementary material for: Reconstruction of gene regulatory networks reveals chromatin remodelers and key transcription factors in tumorigenesis
Source: Genome Med. 2016 May 19;8:57. doi: 10.1186/s13073-016-0310-3 (PMC4872343; doi:10.1186/s13073-016-0310-3)
Supplement: Additional file 4: Figure S4. — Gene expression levels of chromatin remodelers/modulators (CRMs) differentially expressed in the stepwise tumorigenesis system. CRMs are specified on the left together with the corresponding co-expression pathways. The heatmap shows the ratio of expression in BJEL or BJELM cells relative to the expression in BJ cells. The corresponding color code is shown on the right. (PDF 2.95 mb) [file 13073_2016_310_MOESM4_ESM.pdf]

HGNC ID	Chromatin Remodeler/ Modulator	Co-path
4861	HELLS	i
6518	LBR	i
10581	SCML2	i
17287	SUV39H2	i
29535	MTF2	i
5401	SP110	ii
25498	KDM4D	ii
30831	TDRD7	iii
20626	CHD7	iv
367	AKAP1	iv
1915	CHD1	iv
18241	PRMT6	iv
4667	GTF3C4	iv
10894	PRMT5	iv
30163	PRMT3	iv
19441	MINA	iv
4201	KAT2A	iv
19336	SHPRH	iv
29079	KDM1A	iv
12556	UHRF1	iv
11098	SMARCA2	v
20444	MBD5	v
9346	PRDM1	vii
14068	HDAC5	vii

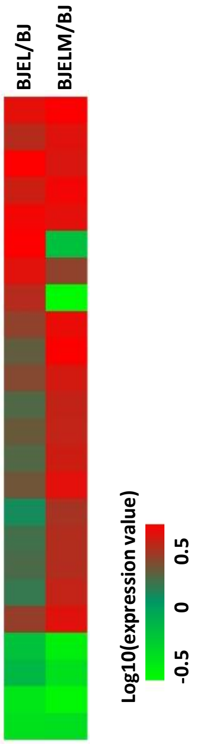

Figure S4. Gene expression levels of Chromatin Remodelers/Modulators (CRM) differentially expressed in the stepwise tumorigenesis system. CRMs are specified on the left together with the corresponding co-expression paths. The heatmap shows the ratio of expression in BJEL or BJELM relatively to the expression in BJ. The corresponding color code is shown on the right.
