# Supplementary material for: Reconstruction of gene regulatory networks reveals chromatin remodelers and key transcription factors in tumorigenesis
Source: Genome Med. 2016 May 19;8:57. doi: 10.1186/s13073-016-0310-3 (PMC4872343; doi:10.1186/s13073-016-0310-3)
Supplement: Additional file 5: Table S1. — Chromatin remodelers/modulators differentially expressed during cell transformation. The table describes their reported function and involvement in cancer and provides the corresponding references (listed in Additional file 6). Abbreviations: BRD bromodomain, CHD chromodomain, HAT histoneacetyltransferases, HMT histonemethyltransferase, TDRD Tudor domain. (PDF 563 kb) [file 13073_2016_310_MOESM5_ESM.pdf]

Supplementary Table S1. Chromatin Remodelers/Modulators differentially expressed during cell transformation. The Table describes their reported function and involvement in cancer, and provides the corresponding references. Abbreviations: TDRD – Tudor domain, HMT – histone methyltransferase, BRD – bromodomain, CHD – chromodomain, HAT – histone acetyltransferases. Supplementary data references are in the Additional file 6 of this manuscript.

**CRM (Alternative symbol) | NCBI gene ID | gene name**			**co-path**	**CR class**	**Functional group / Function**	**Involvement in cancer**
HELLS (LSH)	**3070**	helicase, lymphoid-specific	i	remodeler	SNF2-like protein/ transcriptional repressor [1,2,3]	Overexpressed in prostate cancer; drives retinoblastoma tumor progression [4,5]
LBR	**3930**	lamin B receptor	i	reader	TDRD containing protein/ bind H4K20me2/ Represses trsanscription [6]	Unknown
SCML2	**10389**	sex comb on midleg-like 2	i	-	Polycomb group (PcG) gene, part of PRC1 complex/ binds and modulates CDK/CYCLIN/p21 complexes [7]	Overexpressed in AML patients [8,9]
SUV39H2 (KMT1B)	**79723**	suppressor of variegation 3-9 homolog 2 (Drosophila)	i	writer	HMT/ Trimethylates 'Lys-9' of histone H3/ maintains heterochromatin, ensures genome stability [10,11]	Overexpressed in human lung, bladder cancers, multiple myeloma; suggested a s a novel target of anticancer therapy [12]
MTF2 (PCL2)	**22823**	metal response element binding transcription factor 2	i	-	PcG gene, associates with the PRC2 complex and promotes H3K27me3 at target genes [13]	Unknown
SP110 (IFI45)	**3431**	SP110 nuclear body protein	ii	reader	BRD containing protein, known to recognize acteyl-lysine residues/ potentially involved in chromatin remodeling [14]	Unknown
KDM4D (JMJD2D)	**55693**	lysine (K)-specific demethylase 4D	ii	eraser	Histone demethylase/ demethylase both H3K9me2 and H3K9me3 [15]	Both pro-oncogenic function and activation of p53-dependent gene expression are reported [16]
TDRD7	**23424**	tudor domain containing 7	iii	reader	TDRD containing protein/ preferentially binds H3K9me2/3 [17]	Unknown
CHD7	**55636**	chromodomain helicase DNA binding protein 7	iv	reader	CHD enzyme of SNF2 family/ bind to methylated H3K4/ positive regulator of rRNA biogenesis [18,19]	Dysregulated in 13-35% of pancreatic adenocarcinoma; low CHD7 expression predicts higher survival outcomes in patients with pancreatic cancer [20,21]
AKAP1 (TDRD17)	**8165**	A kinase (PRKA) anchor protein 1	iv	reader	Protein, containing TDRD, known to bind di- and trimethylated lysine and dimethylated arginine [22]	Unknown
CHD1	**1105**	chromodomain helicase DNA binding protein 1	iv	reader	CHD, containing protein/ Bind trimethylated lysine with sequence specificity via tandem chromodomain [23]	Tumor suppressor required for ERG rearrangement in prostate cancer [24]
PRMT6	**55170**	protein arginine methyltransferase 6	iv	writer	Methyltransferase/ Generates monomethylarginine and asymmetric dimethylarginine [25,26]	Knockdown of PRMT6 inosteosarcoma cells led to the up-regulation of THBS1, natural inhibitor of angiogenesis adn cell migration [27]

CRM (Alternative symbol) | NCBI gene ID | gene name			co-path	CR class	Functional group / Function	Involvement in cancer
GTF3C4 (KAT12)	**9329**	general transcription factor IIIC, polypeptide 4, 90kDa	iv	writer	HAT/ has substrate specificity for H3 [28]	Unknown
PRMT5	**10419**	protein arginine methyltransferase 5	iv	writer	Generates mono- and dimethylarginin, dimethyates histones H3 and H4 and nonchromatin proteins [29,30]	Overexpressed in ovarian cancer and glioblastoma, candidate therapeutic target in glioblastoma [31,32]
PRMT3	**10196**	protein arginine methyltransferase 3	iv	writer	Generates monomethylarginine and asymmetric dimethylarginine [33]	Unknown
MINA (MDIG)	**84864**	MYC induced nuclear antigen	iv	eraser	Histone demethylase/ demethylase of H3K9me3 [34,35]	Overexpressed in colon cancer, associated with lung cancer, potential prognostic factor for esophageal squamous cell carcinoma; marker of adenocarcinoma [36-39]
KAT2A (GCN5)	**2648**	K(Lysine) acetyltransferase 2A	iv	writer	HAT/ belongs to SAGA and ATAC complexes [40,41]	Promote transcription of TNFRSF10A/10B in lung cancer cells [42]
SHPRH	**257218**	SNF2 histone linker PHD RING helicase	iv	reader?	PhD containing protein, but doesn't recognize H3K4/ Poly-Ubiquitinate PCNA [43]	Possible candidate for the tumor suppressor gene [44]
KDM1A (KDM1A)	**23028**	lysine (K)-specific demethylase 1	iv	eraser	HMT/ Demethylate both H3K4me1/me2 and H3K9me1/me2, thus acting as coactivator or a corepressor [45,46]	Overexpressed in bladder, colorectal, gastric, lung, ER-negative breast cancers, chondrosarcoma, osteosarcoma [47-49]
UHRF1	**29128**	ubiquitin-like with PHD and RING finger domains 1	iv	reader	TDRD and PHD containing protein/ Binds to H3K9me3 and unmodified H3 [50,51]	Drive retinoblastoma tumor progression; upregulated and associated with cellular proliferation on colorectal cancer; overexpressed in hepatocellular carcinoma [5,52,53]
SMARCA2 (HBRM)	**6595**	SWI/SNF related, matrix associated, actin dep-t regulator of chromatin A2	v	reader	BRD containing protein/ stabilize the interaction between DNA and histones [54]	Loss of SMARCA2 confer a proliferative advantage in prostate cancer [55]
MBD5	**55777**	methyl-CpG binding domain protein 5	v	reader	Binds to heterochromatin, but does not interact with methylated or unmethylated DNA [56]	Unknown
PRDM1	**639**	positive regulatory domain-containing protein 1	vii	writer	Methyltransferase/ potential nucleator for H3K27me3 accumulation and spread [22,57]	Tumor suppressor in diffuse large B-cell lymphomas, in aggressie natural killer cell lymphomas and anaplastic large-cell lymphoma [58-60]
HDAC5	**10014**	histone deacetylase 5	vii	eraser	Deacetylase/ required for maintenance of pericentric heterochromatin and long telomeres [61,62]	Downregulated in glioblastoma [63]
