# Supplementary material for: Reconstruction of gene regulatory networks reveals chromatin remodelers and key transcription factors in tumorigenesis
Source: Genome Med. 2016 May 19;8:57. doi: 10.1186/s13073-016-0310-3 (PMC4872343; doi:10.1186/s13073-016-0310-3)
Supplement: Additional file 6. — Supplementary data references. (DOCX 21 kb) [file 13073_2016_310_MOESM6_ESM.docx]

**Supplementary data references**

1. Jarvis, C. D. *et al.* A novel putative helicase produced in early murine lymphocytes. *Gene* **169,** 203–207 (1996).

2. Dennis, K., Fan, T., Geiman, T., Yan, Q. & Muegge, K. Lsh, a member of the SNF2 family, is required for genome-wide methylation. *Genes Dev.* **15,** 2940–2944 (2001).

3. Myant, K. & Stancheva, I. LSH cooperates with DNA methyltransferases to repress transcription. *Mol. Cell. Biol.* **28,** 215–226 (2008).

4. Von Eyss, B. *et al.* The SNF2-like helicase HELLS mediates E2F3-dependent transcription and cellular transformation. *EMBO J.* **31,** 972–985 (2011).

5. Benavente, C. A., Finkelstein, D., Johnson, D. A., Ashery-padan, R. & Dyer, M. A. Chromatin remodelers HELLS and UHRF1 mediate the epigenetic deregulation of genes that drive retinoblastoma tumor progression. *Oncotarget* **5,** 9594–9608 (2014).

6. Hirano, Y. *et al.* Lamin B receptor recognizes specific modifications of histone H4 in heterochromatin formation. *J. Biol. Chem.* **287,** 42654–42663 (2012).

7. Lecona, E. *et al.* Polycomb Protein SCML2 Regulates the Cell Cycle by Binding and Modulating CDK/CYCLIN/p21 Complexes. *PLoS Biol.* **11,** (2013).

8. Valk, P. J. M. *et al.* Prognostically useful gene-expression profiles in acute myeloid leukemia. *N. Engl. J. Med.* **350,** 1617–1628 (2004).

9. Grubach, L. *et al.* Gene expression profiling of Polycomb, Hox and Meis genes in patients with acute myeloid leukaemia. *Eur. J. Haematol.* **81,** 112–122 (2008).

10. Peters, A. H. F. M. *et al.* Loss of the Suv39h histone methyltransferases impairs mammalian heterochromatin and genome stability. *Cell* **107,** 323–337 (2001).

11. García-Cao, M., O’Sullivan, R., Peters, A. H. F. M., Jenuwein, T. & Blasco, M. a. Epigenetic regulation of telomere length in mammalian cells by the Suv39h1 and Suv39h2 histone methyltransferases. *Nat. Genet.* **36,** 94–99 (2004).

12. Sone, K. *et al.* Critical role of lysine 134 methylation on histone H2AX for γ-H2AX production and DNA repair. *Nat. Commun.* **5,** 5691 (2014).

13. Walker, E. *et al.* Polycomb-like 2 Associates with PRC2 and Regulates Transcriptional Networks during Mouse Embryonic Stem Cell Self-Renewal and Differentiation. *Cell Stem Cell* **6,** 153–166 (2010).

14. Cai, L., Wang, Y., Wang, J.-F. & Chou, K.-C. Identification of proteins interacting with human SP110 during the process of viral infections. *Med. Chem.* **7,** 121–126 (2011).

15. Whetstine, J. R. *et al.* Reversal of Histone Lysine Trimethylation by the JMJD2 Family of Histone Demethylases. *Cell* **125,** 467–481 (2006).

16. Kim, T.-D., Oh, S., Shin, S. & Janknecht, R. Regulation of Tumor Suppressor p53 and HCT116 Cell Physiology by Histone Demethylase JMJD2D/KDM4D. *PLoS One* **7,** e34618 (2012).

17. Bua, D. J. *et al.* Epigenome microarray platform for proteome-wide dissection of chromatin-signaling networks. *PLoS One* **4,** (2009).

18. Schnetz, M. P. *et al.* Genomic distribution of CHD7 on chromatin tracks H3K4 methylation patterns. *Genome Res.* **19,** 590–601 (2009).

19. Zentner, G. E. *et al.* CHD7 functions in the nucleolus as a positive regulator of ribosomal RNA biogenesis. *Hum. Mol. Genet.* **19,** 3491–3501 (2010).

20. Colbert, L. E. *et al.* CHD7 expression predicts survival outcomes in patients with resected pancreatic cancer. *Cancer Res.* **74,** 2677–2687 (2014).

21. Cerami, E. *et al.* The cBio Cancer Genomics Portal: An open platform for exploring multidimensional cancer genomics data. *Cancer Discov.* **2,** 401–404 (2012).

22. Arrowsmith, C. H., Bountra, C., Fish, P. V., Lee, K. & Schapira, M. Epigenetic protein families: a new frontier for drug discovery. *Nat. Rev. Drug Discov.* **11,** 384–400 (2012).

23. Sims, R. J. *et al.* Human but not yeast CHD1 binds directly and selectively to histone H3 methylated at lysine 4 via its tandem chromodomains. *J. Biol. Chem.* **280,** 41789–41792 (2005).

24. Burkhardt, L. *et al.* CHD1 Is a 5q21 tumor suppressor required for ERG rearrangement in prostate cancer. *Cancer Res.* **73,** 2795–2805 (2013).

25. Frankel, A. *et al.* The novel human protein arginine N-methyltransferase PRMT6 is a nuclear enzyme displaying unique substrate specificity. *J. Biol. Chem.* **277,** 3537–3543 (2002).

26. Guccione, E. *et al.* Methylation of histone H3R2 by PRMT6 and H3K4 by an MLL complex are mutually exclusive. *Nature* **449,** 933–937 (2007).

27. Michaud-Levesque, J. & Richard, S. Thrombospondin-1 is a transcriptional repression target of PRMT6. *J. Biol. Chem.* **284,** 21338–21346 (2009).

28. Hsieh, Y. J., Kundu, T. K., Wang, Z., Kovelman, R. & Roeder, R. G. The TFIIIC90 subunit of TFIIIC interacts with multiple components of the RNA polymerase III machinery and contains a histone-specific acetyltransferase activity. *Mol. Cell. Biol.* **19,** 7697–7704 (1999).

29. Dacwag, C. S., Ohkawa, Y., Pal, S., Sif, S. & Imbalzano, A. N. The protein arginine methyltransferase Prmt5 is required for myogenesis because it facilitates ATP-dependent chromatin remodeling. *Mol. Cell. Biol.* **27,** 384–394 (2007).

30. Ren, J. *et al.* Methylation of ribosomal protein S10 by protein-arginine methyltransferase 5 regulates ribosome biogenesis. *J. Biol. Chem.* **285,** 12695–12705 (2010).

31. Bao, X. *et al.* Overexpression of PRMT5 promotes tumor cell growth and is associated with poor disease prognosis in epithelial ovarian cancer. *J. Histochem. Cytochem.* **61,** 206–17 (2013).

32. Yan, F. *et al.* Genetic validation of the protein arginine methyltransferase PRMT5 as a candidate therapeutic target in glioblastoma. *Cancer Res.* **74,** 1752–1765 (2014).

33. Bachand, F. & Silver, P. a. PRMT3 is a ribosomal protein methyltransferase that affects the cellular levels of ribosomal subunits. *EMBO J.* **23,** 2641–2650 (2004).

34. Lu, Y. *et al.* Lung cancer-associated JmjC domain protein mdig suppresses formation of tri-methyl lysine 9 of histone H3. *Cell Cycle* **8,** 2101–2109 (2009).

35. Tsuneoka, M., Koda, Y., Soejima, M., Teye, K. & Kimura, H. A novel Myc target gene, mina53, that is involved in cell proliferation. *J. Biol. Chem.* **277,** 35450–35459 (2002).

36. Zhang, Y. *et al.* The Human mineral dust-induced gene, mdig, is a cell growth regulating gene associated with lung cancer. *Oncogene* **24,** 4873–4882 (2005).

37. Teye, K. *et al.* Increased expression of a Myc target gene Mina53 in human colon cancer. *Am. J. Pathol.* **164,** 205–216 (2004).

38. Tsuneoka, M. *et al.* Mina53 as a potential prognostic factor for esophageal squamous cell carcinoma. *Clin. Cancer Res.* **10,** 7347–7356 (2004).

39. Xing, J., Wang, K., Liu, P.-W., Miao, Q. & Chen, X.-Y. Mina53, a novel molecular marker for the diagnosis and prognosis of gastric adenocarcinoma. *Oncol. Rep.* **31,** 634–640 (2014).

40. Burgess, R. J. & Zhang, Z. Roles for Gcn5 in promoting nucleosome assembly and maintaining genome integrity. *Cell Cycle* **9,** 2979–2985 (2010).

41. Atanassov, B. S. *et al.* NIH Public Access. **35,** 352–364 (2010).

42. Li, T. *et al.* DDIT3 and KAT2A Proteins Regulate TNFRSF10A and TNFRSF10B Expression in Endoplasmic Reticulum Stress-mediated Apoptosis in Human Lung Cancer Cells. *J. Biol. Chem.* **290,** 11108–11118 (2015).

43. Motegi, A. *et al.* Human SHPRH suppresses genomic instability through proliferating cell nuclear antigen polyubiquitination. *J. Cell Biol.* **175,** 703–708 (2006).

44. Sood, R. *et al.* Cloning and characterization of a novel gene, SHPRH, encoding a conserved putative protein with SNF2/helicase and PHD-finger domains from the 6q24 region. *Genomics* **82,** 153–161 (2003).

45. Shi, Y. *et al.* Histone demethylation mediated by the nuclear amine oxidase homolog LSD1. *Cell* **119,** 941–953 (2004).

46. Metzger, E. *et al.* LSD1 demethylates repressive histone marks to promote androgen-receptor-dependent transcription. *Nature* **437,** 436–439 (2005).

47. Hayami, S. *et al.* Overexpression of LSD1 contributes to human carcinogenesis through chromatin regulation in various cancers. *Int. J. Cancer* **128,** 574–586 (2011).

48. Lim, S. *et al.* Lysine-specific demethylase 1 (LSD1) is highly expressed in ER-negative breast cancers and a biomarker predicting aggressive biology. *Carcinogenesis* **31,** 512–520 (2010).

49. Schulte, J. H. *et al.* Lysine-specific demethylase 1 is strongly expressed in poorly differentiated neuroblastoma: Implications for therapy. *Cancer Res.* **69,** 2065–2071 (2009).

50. Nady, N. *et al.* Recognition of multivalent histone states associated with heterochromatin by UHRF1 protein. *J. Biol. Chem.* **286,** 24300–24311 (2011).

51. Lallous, N. *et al.* The PHD finger of human UHRF1 reveals a new subgroup of unmethylated histone H3 tail readers. *PLoS One* **6,** (2011).

52. Kofunato, Y. *et al.* UHRF1 expression is upregulated and associated with cellular proliferation in colorectal cancer. *Oncol. Rep.* **28,** 1997–2002 (2012).

53. Mudbhary, R. *et al.* UHRF1 Overexpression Drives DNA Hypomethylation and Hepatocellular Carcinoma. *Cancer Cell* **25,** 196–209 (2014).

54. Schnitzler, G., Sif, S. & Kingston, R. E. Human SWI/SNF interconverts a nucleosome between its base state and a stable remodeled state. *Cell* **94,** 17–27 (1998).

55. Shen, H. *et al.* The SWI/SNF ATPase Brm is a gatekeeper of proliferative control in prostate cancer. *Cancer Res.* **68,** 10154–10162 (2008).

56. Laget, S. *et al.* The human proteins MBD5 and MBD6 associate with heterochromatin but they do not bind methylated DNA. *PLoS One* **5,** (2010).

57. Kurimoto, K. *et al.* Quantitative Dynamics of Chromatin Remodeling during Germ Cell Specification from Mouse Embryonic Stem Cells. *Cell Stem Cell* **16,** 517–532 (2015).

58. Tam, W. *et al.* Mutational analysis of PRDM1 indicates a tumor-suppressor role in diffuse large B-cell lymphomas. *Blood* **107,** 4090–4100 (2006).

59. Kucuk, C. *et al.* PRDM1 is a tumor suppressor gene in natural killer cell malignancies. *Proc. Natl. Acad. Sci.* **108,** 20119–20124 (2011).

60. Boi, M. *et al.* PRDM1/BLIMP1 is commonly inactivated in anaplastic large T-cell lymphoma. *Blood* **122,** 2683–93 (2013).

61. Peixoto, P. *et al.* HDAC5 is required for maintenance of pericentric heterochromatin, and controls cell-cycle progression and survival of human cancer cells. *Cell Death Differ.* **19,** 1239–1252 (2012).

62. Novo, C. L. *et al.* A new role for histone deacetylase 5 in the maintenance of long telomeres. *FASEB J.* **27,** 3632–3642 (2013).

63. Lucio-Eterovic, A. K. B. *et al.* Differential expression of 12 histone deacetylase (HDAC) genes in astrocytomas and normal brain tissue: class II and IV are hypoexpressed in glioblastomas. *BMC Cancer* **8,** 243 (2008).
