# Supplementary material for: Reconstruction of gene regulatory networks reveals chromatin remodelers and key transcription factors in tumorigenesis
Source: Genome Med. 2016 May 19;8:57. doi: 10.1186/s13073-016-0310-3 (PMC4872343; doi:10.1186/s13073-016-0310-3)
Supplement: Additional file 7: Table S2. — Transcription factors preferentially associated with specific co-expression pathways and which originate from deregulated gene programming during tumorigenesis. Some of these TFs are differentially expressed as well and the co-expression pathway they belong to is shown in the last column. (PDF 102 kb) [file 13073_2016_310_MOESM7_ESM.pdf]

**Supplementary Table S2. TFs, preferentially associated with specific co-expression paths**, that originate from deregulated gene programming during tumorigenesis. Some of these TFs are differentially expressed as well, and the co-expression path they belong to is precised in the last column.

| Symbol | NCBI gene ID | Gene_Name | expression ratio BJEL/BJ | expression ratio BJELM/BJ | Co-expression path |
|---|---|---|---|---|---|
| ***DDX58*** | 23586 | DEAD (Asp-Glu-Ala-Asp) box polypeptide 58 | 11,19 | 0,57 | 2 |
| ***TRIM21*** | 6737 | tripartite motif containing 21 | 2,48 | 0,56 | neutral |
| ***SP100*** | 6672 | SP100 nuclear antigen | 2,64 | 0,66 | neutral |
| ***NLRC5*** | 84166 | NLR family, CARD domain containing 5 | 1,65 | 0,98 | neutral |
| ***SP110*** | 3431 | SP110 nuclear body protein | 4,47 | 0,63 | 2 |
| ***IRF1*** | 3659 | interferon regulatory factor 1 | 1,83 | 0,95 | neutral |
| ***IFI16*** | 3428 | interferon, gamma-inducible protein 16 | 1,55 | 0,72 | neutral |
| ***STAT1*** | 6772 | signal transducer and activator of transcription 1, 91kDa | 2,31 | 0,33 | 3 |
| ***STAT6*** | 6778 | signal transducer and activator of transcription 6, interleukin-4 induced | 0,76 | 0,64 | neutral |
| ***IRF7*** | 3665 | interferon regulatory factor 7 | 1,94 | 0,80 | neutral |
| ***ZFP82*** | 284406 | ZFP82 zinc finger protein | 1,58 | 1,32 | neutral |
| ***ZNF876P*** | 642280 | zinc finger protein 876, pseudogene | 2,24 | 1,04 | 2 |
| ***RBCK1*** | 10616 | RanBP-type and C3HC4-type zinc finger containing 1 | 1,08 | 0,57 | neutral |
| ***IRF2*** | 3660 | interferon regulatory factor 2 | 1,54 | 0,53 | neutral |
| ***KDM5C*** | 8242 | lysine (K)-specific demethylase 5C | 1,10 | 0,98 | neutral |
| ***FOXH1*** | 8928 | forkhead box H1 | 0,98 | 0,97 | neutral |
| ***ELF4*** | 2000 | E74-like factor 4 (ets domain transcription factor) | 1,30 | 0,81 | neutral |
| ***HCLS1*** | 3059 | hematopoietic cell-specific Lyn substrate 1 | 1,26 | 1,17 | neutral |
| ***RUNX3*** | 864 | runt-related transcription factor 3 | 0,98 | 1,20 | neutral |
| ***FLI1*** | 2313 | Fli-1 proto-oncogene, ETS transcription factor | 0,99 | 0,44 | 5 |
| ***TEAD4*** | 7004 | TEA domain family member 4 | 1,43 | 2,41 | 4 |
| ***IRF6*** | 3664 | interferon regulatory factor 6 | 0,81 | 0,85 | neutral |
| ***NFIA*** | 4774 | nuclear factor I/A | 0,84 | 2,55 | 4 |
| ***SATB2*** | 23314 | SATB homeobox 2 | 0,88 | 0,73 | neutral |
| ***SMAD7*** | 4092 | SMAD family member 7 | 0,46 | 0,55 | 6 |
| ***STAT2*** | 6773 | signal transducer and activator of transcription 2, 113kDa | 1,77 | 0,24 | 5 |
| ***TEAD1*** | 7003 | TEA domain family member 1 (SV40 transcriptional enhancer factor) | 0,78 | 0,61 | neutral |
| ***PRDM1*** | 639 | PR domain containing 1, with ZNF domain | 0,41 | 0,34 | 7 |
| ***TBX5*** | 6910 | T-box 5 | 0,55 | 0,57 | neutral |
| ***TEAD3*** | 7005 | TEA domain family member 3 | 0,66 | 0,51 | neutral |
| ***MYOCD*** | 93649 | myocardin | 0,18 | 0,10 | 7 |
| ***SOX7*** | 83595 | SRY (sex determining region Y)-box 7 | 0,90 | 0,97 | neutral |
| ***HIC1*** | 3090 | hypermethylated in cancer 1 | 0,82 | 0,84 | neutral |
| ***TBX2*** | 6909 | T-box 2 | 0,69 | 0,69 | neutral |
| ***FOXF2*** | 2295 | forkhead box F2 | 0,65 | 0,60 | neutral |
| ***OSR2*** | 116039 | odd-skipped related transciption factor 2 | 0,44 | 0,27 | 7 |
| ***TBX3*** | 6926 | T-box 3 | 0,70 | 0,78 | neutral |
| ***SNAI2*** | 6591 | snail family zinc finger 2 | 0,86 | 0,65 | neutral |
| ***YAP1*** | 10413 | Yes-associated protein 1 | 0,87 | 0,76 | neutral |
| ***LMO4*** | 8543 | LIM domain only 4 | 0,94 | 0,71 | neutral |
| ***AFF3*** | 3899 | AF4/FMR2 family, member 3 | 0,83 | 0,60 | neutral |
| ***GATA6*** | 2627 | GATA binding protein 6 | 1,16 | 1,42 | neutral |
| ***TFAP2C*** | 7022 | transcription factor AP-2 gamma (activating enhancer binding protein 2 gamma) | 1,15 | 0,26 | 5 |

| Symbol | NCBI gene ID | Gene_Name | expression ratio BJEL/BJ | expression ratio BJELM/BJ | Co-expression path |
|---|---|---|---|---|---|
| ***TBX18*** | 9096 | T-box 18 | 1,25 | 0,90 | neutral |
| ***TRPS1*** | 7227 | trichorhinophalangeal syndrome I | 0,61 | 0,72 | neutral |
| ***HOXA5*** | 3202 | homeobox A5 | 1,33 | 0,99 | neutral |
| ***LHX9*** | 56956 | LIM homeobox 9 | 0,85 | 0,25 | 5 |
| ***SMARCA1*** | 6594 | SWI/SNF related, matrix associated, actin dependent regulator of chromatin, subfamily a, member 1 | 1,00 | 0,57 | neutral |
| ***TSHZ3*** | 57616 | teashirt zinc finger homeobox 3 | 0,75 | 0,37 | 5 |
| ***NDN*** | 4692 | necdin, melanoma antigen (MAGE) family member | 0,87 | 0,69 | neutral |
| ***NR2F2*** | 7026 | nuclear receptor subfamily 2, group F, member 2 | 1,24 | 1,25 | neutral |
| ***HOXC6*** | 3223 | homeobox C6 | 1,38 | 0,74 | neutral |
| ***NFIB*** | 4781 | nuclear factor I/B | 9,92 | 5,52 | 1 |
| ***GLIS3*** | 169792 | GLIS family zinc finger 3 | 0,96 | 1,12 | neutral |
| ***HOXA13*** | 3209 | homeobox A13 | 0,61 | 0,69 | neutral |
| ***JDP2*** | 122953 | Jun dimerization protein 2 | 0,80 | 0,49 | neutral |
| ***AEBP1*** | 165 | AE binding protein 1 | 0,40 | 0,21 | 7 |
| ***KLF2*** | 10365 | Kruppel-like factor 2 | 0,82 | 0,72 | neutral |
| ***KLF4*** | 9314 | Kruppel-like factor 4 (gut) | 1,12 | 0,90 | neutral |
| ***ARID5B*** | 84159 | AT rich interactive domain 5B (MRF1-like) | 0,68 | 0,35 | 5 |
| ***GLIS2*** | 84662 | GLIS family zinc finger 2 | 0,65 | 0,53 | neutral |
| ***FOXC2*** | 2303 | forkhead box C2 (MFH-1, mesenchyme forkhead 1) | 0,82 | 0,79 | neutral |
| ***FOXD1*** | 2297 | forkhead box D1 | 1,37 | 1,50 | neutral |
| ***MSX1*** | 4487 | msh homeobox 1 | 0,85 | 0,87 | neutral |
| ***FOXC1*** | 2296 | forkhead box C1 | 0,80 | 0,89 | neutral |
| ***TWIST1*** | 7291 | twist family bHLH transcription factor 1 | 1,06 | 0,77 | neutral |
| ***WNT5A*** | 7474 | wingless-type MMTV integration site family, member 5A | 0,33 | 0,23 | 7 |
| ***GLI2*** | 2736 | GLI family zinc finger 2 | 0,75 | 0,65 | neutral |
| ***CHD7*** | 55636 | chromodomain helicase DNA binding protein 7 | 1,92 | 2,91 | 4 |
| ***DNMT3A*** | 1788 | DNA (cytosine-5-)-methyltransferase 3 alpha | 1,02 | 0,93 | neutral |
| ***JUN*** | 3725 | jun proto-oncogene | 1,63 | 1,52 | neutral |
| ***SCML1*** | 6322 | sex comb on midleg-like 1 (Drosophila) | 2,29 | 1,82 | 2 |
| ***E2F2*** | 1870 | E2F transcription factor 2 | 1,44 | 1,04 | neutral |
| ***GFI1B*** | 8328 | growth factor independent 1B transcription repressor | 0,93 | 0,97 | neutral |
| ***KLF9*** | 687 | Kruppel-like factor 9 | 0,53 | 0,62 | neutral |
| ***TNFAIP3*** | 7128 | tumor necrosis factor, alpha-induced protein 3 | 0,86 | 0,59 | neutral |
| ***IRF9*** | 10379 | interferon regulatory factor 9 | 2,05 | 0,30 | neutral |
| ***ZBTB38*** | 253461 | zinc finger and BTB domain containing 38 | 1,06 | 1,08 | neutral |
| ***SET*** | 6418 | SET nuclear oncogene | 1,20 | 1,47 | neutral |
| ***ZNF649*** | 65251 | zinc finger protein 649 | 1,55 | 1,78 | neutral |
| ***TFB1M*** | 51106 | transcription factor B1, mitochondrial | 1,14 | 1,91 | neutral |
| ***APEX1*** | 328 | APEX nuclease (multifunctional DNA repair enzyme) 1 | 1,14 | 1,39 | neutral |
| ***RFC1*** | 5981 | replication factor C (activator 1) 1, 145kDa | 1,82 | 1,98 | neutral |
| ***ZNF146*** | 7705 | zinc finger protein 146 | 1,19 | 1,44 | neutral |
| ***DDX1*** | 1653 | DEAD (Asp-Glu-Ala-Asp) box helicase 1 | 0,96 | 1,18 | neutral |
| ***ILF2*** | 3608 | interleukin enhancer binding factor 2 | 1,32 | 1,32 | neutral |
| ***ZNF473*** | 25888 | zinc finger protein 473 | 2,34 | 2,74 | 1 |
| ***HNRNPAB*** | 3182 | heterogeneous nuclear ribonucleoprotein A/B | 1,31 | 1,67 | neutral |
| ***RUVBL2*** | 10856 | RuvB-like AAA ATPase 2 | 1,52 | 2,25 | 4 |

| Symbol | NCBI gene ID | Gene_Name | expression ratio BJEL/BJ | expression ratio BJELM/BJ | Co-expression path |
|---|---|---|---|---|---|
| ***ZNF114*** | 163071 | zinc finger protein 114 | 1,52 | 1,13 | neutral |
| ***TET1*** | 80312 | tet methylcytosine dioxygenase 1 | 0,80 | 1,02 | neutral |
| ***ZIK1*** | 284307 | zinc finger protein interacting with K protein 1 | 2,48 | 1,31 | 2 |
| ***HMGB2*** | 3148 | high mobility group box 2 | 2,10 | 2,24 | neutral |
| ***PURA*** | 5813 | purine-rich element binding protein A | 0,92 | 0,87 | neutral |
| ***ZNF641*** | 121274 | zinc finger protein 641 | 0,74 | 0,42 | neutral |
| ***ZNF93*** | 81931 | zinc finger protein 93 | 4,45 | 2,34 | 1 |
| ***BRIP1*** | 83990 | BRCA1 interacting protein C-terminal helicase 1 | 2,71 | 2,68 | neutral |
| ***TOP2A*** | 7153 | topoisomerase (DNA) II alpha 170kDa | 1,78 | 1,88 | neutral |
| ***E2F1*** | 1869 | E2F transcription factor 1 | 1,92 | 1,80 | neutral |
| ***FOXM1*** | 2305 | forkhead box M1 | 1,62 | 1,64 | neutral |
| ***SMARCC1*** | 6599 | SWI/SNF related, matrix associated, actin dependent regulator of chromatin, subfamily c, member 1 | 1,23 | 1,64 | neutral |
| ***BLM*** | 641 | Bloom syndrome, RecQ helicase-like | 3,50 | 6,36 | 1 |
| ***BRCA1*** | 672 | breast cancer 1, early onset | 3,57 | 4,95 | 1 |
| ***ZNF85*** | 7639 | zinc finger protein 85 | 2,40 | 1,58 | 2 |
| ***ORC1*** | 4998 | origin recognition complex, subunit 1 | 3,83 | 4,44 | neutral |
| ***PCNA*** | 5111 | proliferating cell nuclear antigen | 1,69 | 1,96 | neutral |
| ***WHSC1*** | 7468 | Wolf-Hirschhorn syndrome candidate 1 | 1,76 | 1,78 | neutral |
| ***UHRF1*** | 29128 | ubiquitin-like with PHD and ring finger domains 1 | 1,98 | 2,78 | 4 |
| ***TMPO*** | 7112 | thymopoietin | 2,23 | 2,44 | neutral |
| ***ZNF215*** | 7762 | zinc finger protein 215 | 1,46 | 1,55 | neutral |
| ***MYBL2*** | 4605 | v-myb avian myeloblastosis viral oncogene homolog-like 2 | 2,94 | 3,28 | neutral |
| ***ZNF367*** | 195828 | zinc finger protein 367 | 2,27 | 1,71 | neutral |
| ***BRCA2*** | 675 | breast cancer 2, early onset | 3,40 | 4,19 | 1 |
| ***PCGF6*** | 84108 | polycomb group ring finger 6 | 1,69 | 2,00 | neutral |
| ***MCM8*** | 84515 | minichromosome maintenance complex component 8 | 2,31 | 2,97 | neutral |
| ***TFDP1*** | 7027 | transcription factor Dp-1 | 1,35 | 1,70 | neutral |
| ***DEK*** | 7913 | DEK oncogene | 1,63 | 1,56 | neutral |
| ***EZH2*** | 2146 | enhancer of zeste homolog 2 (Drosophila) | 2,13 | 2,08 | neutral |
| ***HELLS*** | 3070 | helicase, lymphoid-specific | 2,81 | 4,63 | 1 |
| ***CDT1*** | 81620 | chromatin licensing and DNA replication factor 1 | 2,40 | 2,66 | 1 |
| ***POLA1*** | 5422 | polymerase (DNA directed), alpha 1, catalytic subunit | 2,47 | 3,24 | 1 |
| ***SAP30*** | 8819 | Sin3A-associated protein, 30kDa | 1,01 | 0,81 | neutral |
| ***CRY2*** | 1408 | cryptochrome circadian clock 2 | 0,93 | 0,89 | neutral |
| ***ZBTB20*** | 26137 | zinc finger and BTB domain containing 20 | 0,54 | 0,35 | 5 |
| ***DNMT3B*** | 1789 | DNA (cytosine-5-)-methyltransferase 3 beta | 1,21 | 1,32 | neutral |
| ***HMGA2*** | 8091 | high mobility group AT-hook 2 | 0,71 | 0,71 | neutral |
| ***ATXN1*** | 6310 | ataxin 1 | 0,55 | 0,37 | 5 |
| ***HMGA1*** | 3159 | high mobility group AT-hook 1 | 0,90 | 1,58 | neutral |
| ***HDAC2*** | 3066 | histone deacetylase 2 | 1,23 | 1,27 | neutral |
| ***TRIM24*** | 8805 | tripartite motif containing 24 | 1,16 | 1,43 | neutral |
| ***TADA2B*** | 93624 | transcriptional adaptor 2B | 0,96 | 0,77 | neutral |
| ***CREBRF*** | 153222 | CREB3 regulatory factor | 0,61 | 0,31 | 5 |
| ***SMARCAD1*** | 56916 | SWI/SNF-related, matrix-associated actin-dependent regulator of chromatin, subfamily a, containing DEAD/H box 1 | 1,23 | 1,47 | neutral |

| Symbol | NCBI gene ID | Gene_Name | expression ratio BJEL/BJ | expression ratio BJELM/BJ | Co-expression path |
|---|---|---|---|---|---|
| ***NONO*** | 4841 | non-POU domain containing, octamer-binding | 1,23 | 1,33 | neutral |
| ***TTF2*** | 8458 | transcription termination factor, RNA polymerase II | 1,86 | 2,27 | neutral |
| ***SSRP1*** | 6749 | structure specific recognition protein 1 | 1,73 | 2,65 | 4 |
| ***MYC*** | 4609 | v-myc avian myelocytomatosis viral oncogene homolog | 1,55 | 0,82 | 4 |
| ***MTERF3*** | 51001 | Mitochondrial Transcription Termination Factor 3 | 1,85 | 2,26 | 4 |
| ***ZBTB9*** | 221504 | zinc finger and BTB domain containing 9 | 1,39 | 1,55 | neutral |
| ***TAF9*** | 6880 | TAF9 RNA polymerase II, TATA box binding protein (TBP)-associated factor, 32kDa | 1,33 | 1,58 | neutral |
| ***DHX33*** | 56919 | DEAH (Asp-Glu-Ala-His) box polypeptide 33 | 1,70 | 3,97 | 4 |
| ***NOLC1*** | 9221 | nucleolar and coiled-body phosphoprotein 1 | 1,98 | 3,76 | neutral |
