# Supplementary material for: Reconstruction of gene regulatory networks reveals chromatin remodelers and key transcription factors in tumorigenesis
Source: Genome Med. 2016 May 19;8:57. doi: 10.1186/s13073-016-0310-3 (PMC4872343; doi:10.1186/s13073-016-0310-3)
Supplement: Additional file 8: Figure S5. — Classification heatmap model showing the loss of fibroblast identity by BJ fibroblasts during the transformation, while gaining traits of embryonic stem cells. The analysis was performed using the CellNet tool. The color key shows the similarity between the training system and study samples. Yellow and black indicate high and low levels of resemblance, respectively. b.r. biological replicate. (PDF 2.96 mb) [file 13073_2016_310_MOESM8_ESM.pdf]

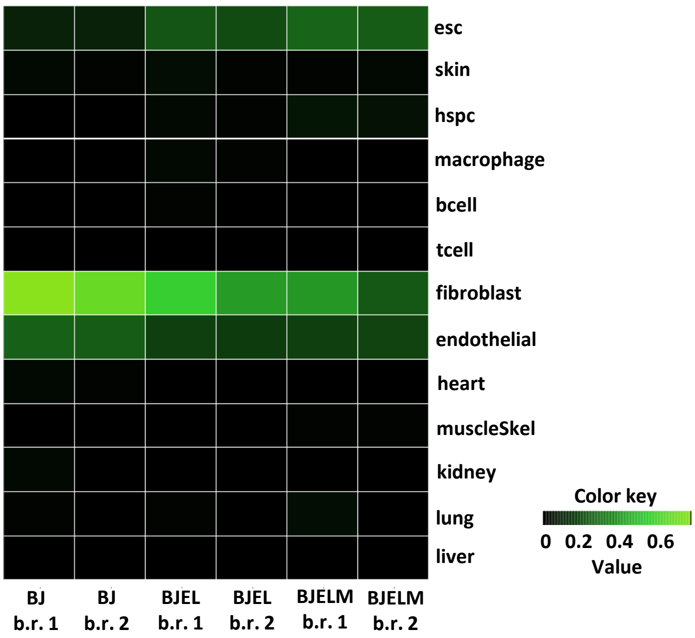

Figure S5. Classification heatmap model showing the loss of fibroblast identity by BJ fibroblasts during the transformation, while gaining traits of embryonic stem cells. Analysis performed using CellNet tool. Color key shows the similarity between the training system and study samples. Yellow and black colors indicate high and low level of resemblance, respectively. *b.r. – biological replicate.
