# Supplementary material for: Reconstruction of gene regulatory networks reveals chromatin remodelers and key transcription factors in tumorigenesis
Source: Genome Med. 2016 May 19;8:57. doi: 10.1186/s13073-016-0310-3 (PMC4872343; doi:10.1186/s13073-016-0310-3)
Supplement: Additional file 9: Figure S6. — A two-step normalization procedure required for proper multiprofile comparison. a To account for technical aspects like antibody efficiency and sequencing depth, we used Epimetheus, a two-step normalization procedure in which (i) the raw count intensity in ChIP-seq datasets produced with antibodies targeting the same factor are corrected following a quantile normalization procedure; then (ii) normalized ChIP-seq profile read counts corresponding to a variety of factors are brought to the same scale via a z-score normalization correction. b The effect of the quantile normalization on H3K9ac datasets assessed in all three cell lines of the stepwise transformation model. Notice that BJELM cells display lower intensity levels of the H3K9ac mark in the LBR promoter (blue arrow) relative to BJ and BJEL cells, while LBR gene expression is upregulated in BJEL and BJELM cells; after quantile correction, the levels in the BJELM dataset appears the same as in the BJEL dataset, both higher than in the BJ dataset. Furthermore, notice the higher background (region under the red brace) in the raw profiles of the BJEL dataset (in comparison with BJ and BJELM), which is brought to the same level in all three datasets after normalization. (PDF 520 kb) [file 13073_2016_310_MOESM9_ESM.pdf]

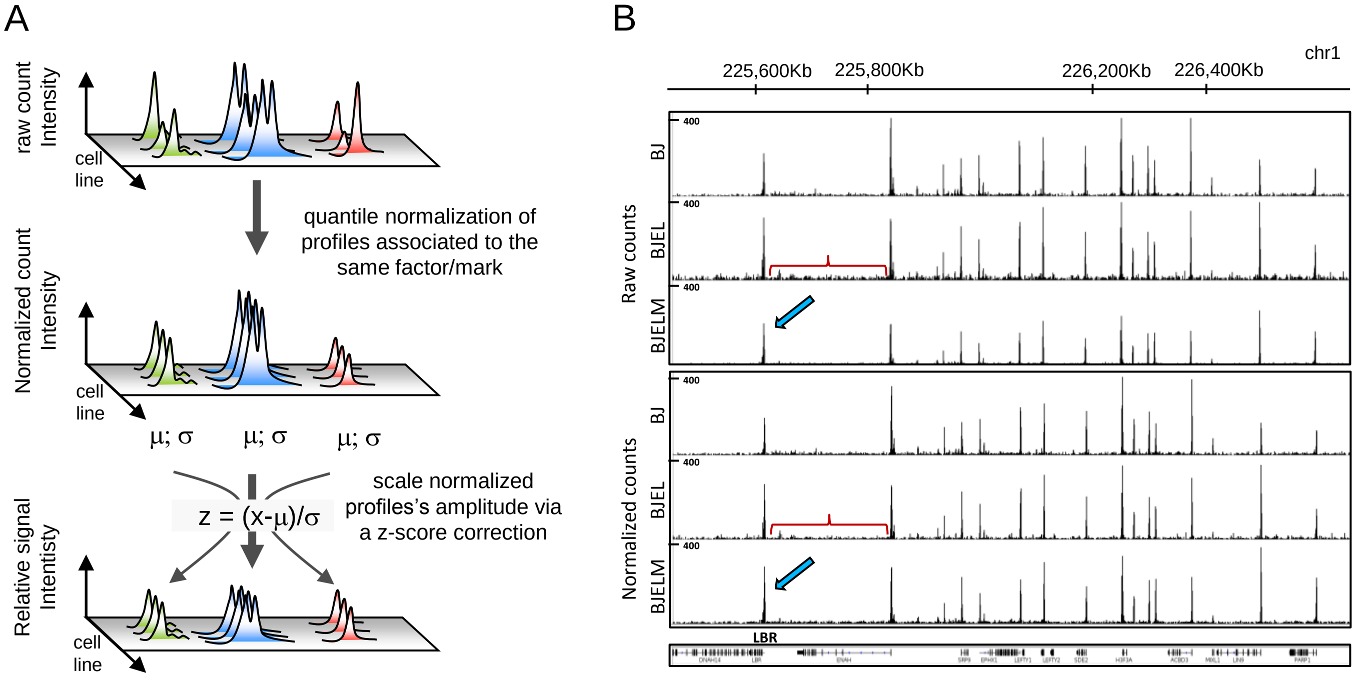

Figure S6. A two-steps normalization procedure required for proper multiprofile comparison. a, To account for technical aspects like antibody efficiency and sequencing depth we used Epimetheus, a two-step normalization procedure in which (i) the raw count intensity in ChIP-seq datasets produced with antibodies targeting the same factor are correcting following a quantile normalization procedure; then (ii) normalized ChIP-seq profiles' read-counts corresponding to a variety of factors are brought to the same scale via a z-score normalization correction. **b,** The effect of the quantile normalization on H3K9ac datasets assessed in all 3 cell lines of stepwise transformation model. Notice that BJELM cells display lower intensity levels of H3K9ac mark in the *LBR* promoter (blue arrow) relative to BJ and BJEL cells, while *LBR* gene expression is upregulated in BJEL and BJELM cells; while after quantile correction, BJELM dataset appears at the same level as BJEL, both higher than in BJ dataset. Furthermore, notice higher background (region under the red brace) in raw profiles of BJEL dataset (in comparison to BJ and BJELM), that is brought to the same level in all three datasets after normalization.
