# Supplementary material for: Reconstruction of gene regulatory networks reveals chromatin remodelers and key transcription factors in tumorigenesis
Source: Genome Med. 2016 May 19;8:57. doi: 10.1186/s13073-016-0310-3 (PMC4872343; doi:10.1186/s13073-016-0310-3)
Supplement: Additional file 11: Figure S7. — Chromatin state annotation. a Statistical analysis of chromatin states (initial and combined) at the TSSs of DEGs. b Chromatin state classification. c Normalized ChIP signal intensities at the TSSs ±500 bp, ordered from first to 26th chromatin state as in a in BJ cells. (PDF 2.95 mb) [file 13073_2016_310_MOESM11_ESM.pdf]

A

Combined chromatin state	Chromatin state (initial)	# of transcripts/chromatin state		
		BJ	BJEL	BJELM
active	1	3223	3178	3504
	2	685	110	201
	3	0	7	2
	4	15	6	18
weakly active	5	5	1	1
	6	36	6	8
	7	12	6	6
transcription prone	8	355	1103	420
	9	532	447	380
	10	1	6	3
	11	73	84	60
bivalent	12	17	6	26
	13	24	28	23
	14	23	5	19
	15	1	2	0
	16	247	113	254
	17	1	0	0
	18	62	54	52
	19	3	9	4
	20	2	0	0
ambiguous	21	22	74	68
	22	90	58	134
	23	77	58	30
	24	22	64	28
empty	25	1982	2076	2217
repressed	26	84	93	136

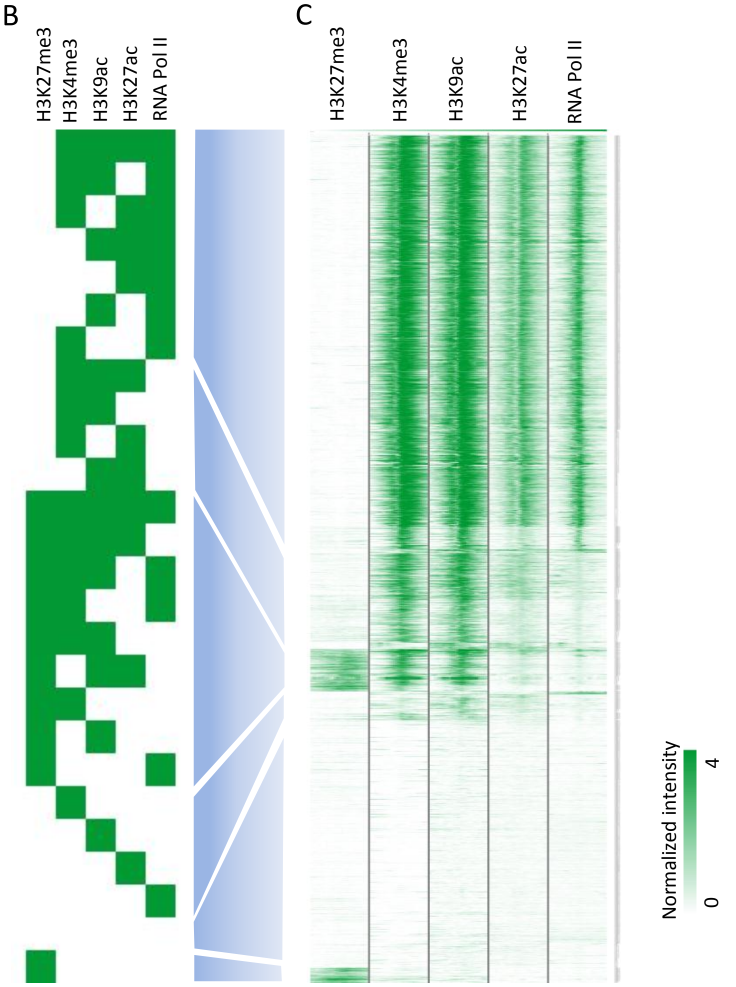

Figure S7. Chromatin state annotation. **a,** Statistical analysis of chromatin states (initial and combined) at the TSSs of DEGs. **b,** Chromatin state classification. Green box stands for the presence of epigenetic mark or polII at the TSSs ± 500bp, white – for the absence. **c,** Normalized ChIP signal intensities at the TSSs ± 500bp, ordered from 1st to 26th chromatin state as in (A) in BJ cells.
