# Supplementary material for: Reconstruction of gene regulatory networks reveals chromatin remodelers and key transcription factors in tumorigenesis
Source: Genome Med. 2016 May 19;8:57. doi: 10.1186/s13073-016-0310-3 (PMC4872343; doi:10.1186/s13073-016-0310-3)
Supplement: Additional file 13: Figure S8. — Cross-cancer alteration summary for CHD7, DHX33, NOLC1, and MYC among 123 cancer types; 99 cancer types that have alterations in these genes are displayed in the histogram. In 49 types of cancer, alterations in these genes occur in more than 10 % of cases. In particular, breast cancer, neuroendocrine prostate cancer, and ovarian serous cystadenocarcinoma have the highest rate of amplification of these genes in tumor samples (55.2, 50.5, and 44.1 % of cases, respectively). (PDF 2990 kb) [file 13073_2016_310_MOESM13_ESM.pdf]

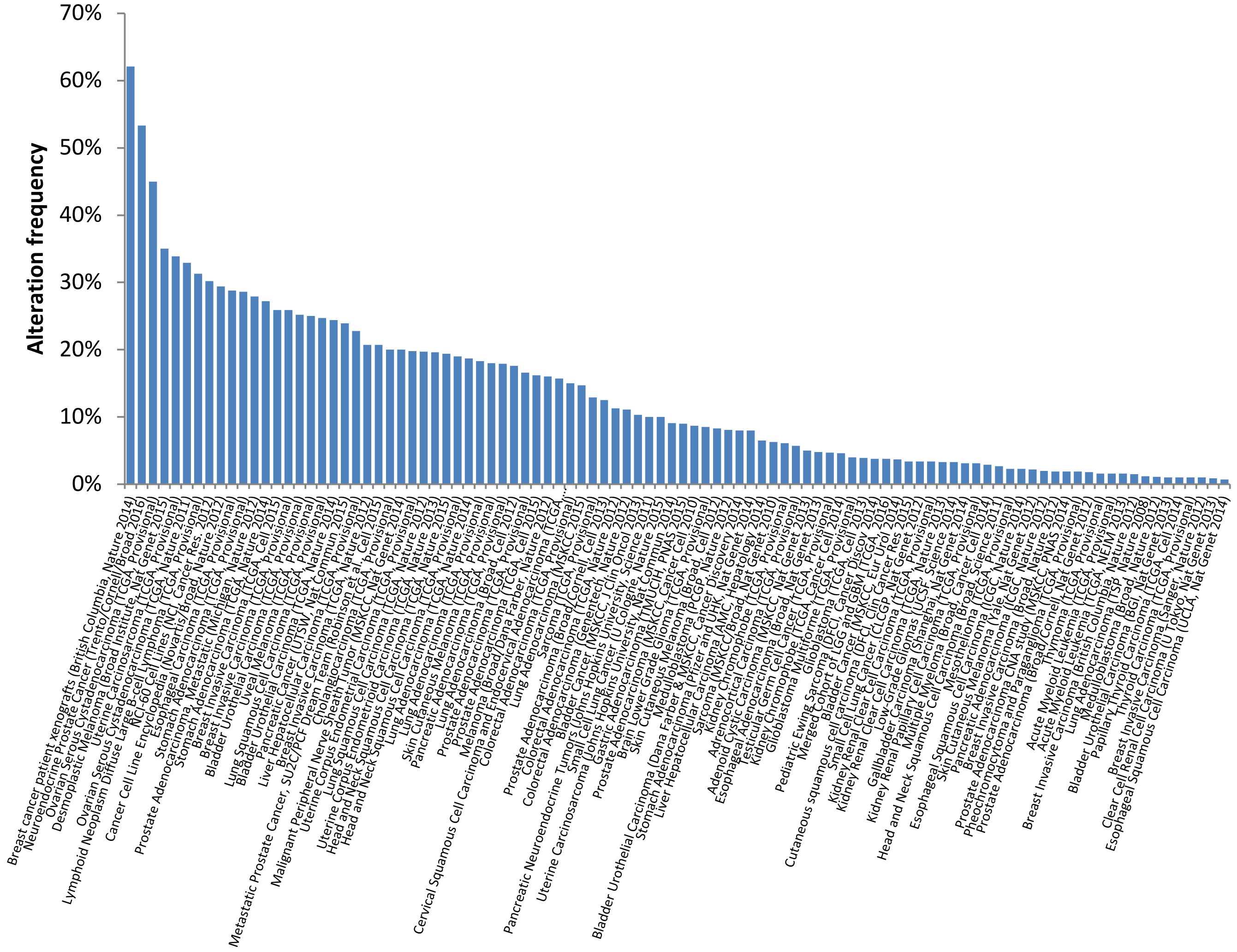

Figure S8. Cross-cancer alteration summary for CHD7,DHX33, NOLC1 and MYC among 123 studies. 99 cancer types, that possess alteration in these genes are displayed in the histogram. In 49 types of cancer alterations in these genes occur in more than 10% of cases In particular, Breast cancer, NEPC (Neuroendocrine Prostate Cancer) and Ovarian Serous Cystadenocarcinoma have the highest rate of alterations of these genes in tumor samples – 62,1%, 53,3% and 45% of cases, respectively.
